# Selective CO_2_ Photoreduction into CH_4_ Triggered by the Synergy between Oxygen Vacancy and Ru Substitution under Near‐Infrared Light Irradiation

**DOI:** 10.1002/advs.202405668

**Published:** 2024-07-09

**Authors:** Jun Li, Xinglong Liu, Xi Wu, Zhongyi Liu, Zaiwang Zhao, Yifeng Liu, Shixue Dou, Yao Xiao

**Affiliations:** ^1^ Henan Institute of Advanced Technology College of Chemistry Zhengzhou University Zhengzhou 450052 P. R. China; ^2^ College of Energy Materials and Chemistry College of Chemistry and Chemical Engineering Inner Mongolia University Hohhot 010070 P. R. China; ^3^ College of Chemistry and Materials Engineering Wenzhou University Wenzhou 325035 P. R. China; ^4^ Institute of Energy Materials Science (IEMS) University of Shanghai for Science and Technology Shanghai 200093 P. R. China

**Keywords:** active hydrogen, CO_2_ photoreduction, near‐infrared light, oxygen vacancies, Ru substitution

## Abstract

Near‐infrared (NIR) light powdered CO_2_ photoreduction reaction is generally restricted to the separation efficiency of photogenerated carriers and the supply of active hydrogen (*H). Herein, the study reports a retrofitting hydrogenated MoO_3‐x_ (H‐MoO_3‐x_) nanosheet photocatalysts with Ru single atom substitution (Ru@H‐MoO_3‐x_) fabricated by one‐step solvothermal method. Experiments together with theoretical calculations demonstrate that the synergistic effect of Ru substitution and oxygen vacancy can not only inhibit the recombination of photogenerated carriers, but also facilitate the CO_2_ adsorption/activation as well as the supply of *H. Compared with H‐MoO_3‐x_, the Ru@H‐MoO_3‐x_ exhibit more favorable formation of *CHO in the process of *CO conversion due to the fast *H generation on electron‐rich Ru sites and transfer to *CO intermediates, leading to the preferential photoreduction of CO_2_ to CH_4_ with high selectivity. The optimized Ru@H‐MoO_3‐x_ exhibits a superior CO_2_ photoreduction activity with CH_4_ evolution rate of 111.6 and 39.0 µmol g_catalyst_^−1^ under full spectrum and NIR light irradiation, respectively, which is 8.8 and 15.0 times much higher than that of H‐MoO_3‐x_. This work provides an in‐depth understanding at the atomic level on the design of NIR responsive photocatalyst for achieving the goal of carbon neutrality.

## Graphical abstract

The electron‐deficient Mo on Ru@H‐MoO_3‐x_ exhibits more favorable formation of *CHO in the process of *CO conversion due to the fast *H generation and transfer to *CO intermediates on electron‐rich Ru sites, leading to the preferential photoreduction of CO_2_ to CH_4_ high selectivity.

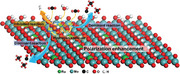

## 1 Introduction

Artificial photosynthesis that can convert of CO_2_ and H_2_O into hydrocarbon fuels has been regarded as an ideal strategy to alleviate the greenhouse effect, as well as fossil energy depletion.^[^ ^1^, ^2^, ^3^, ^4^, ^5^, ^6^, ^7^, ^8^ ^]^ Due to the high C═O dissociation energy of ≈750 kJ mol^−1^ and involved multiple protons coupled electron transfer pathways for CO_2_ photoreduction, the photocatalytic efficiency and selectivity are below expectation.^[^ ^9^, ^10^, ^11^ ^]^ To achieve the high‐efficient photocatalytic activity, the wide bandgap semiconductors of ≈2.0 eV that are required to meet the potentials of CO_2_ reduction and H_2_O oxidation have been widely reported, such as g‐C_3_N_4_, SnS_2_, and BiVO_4_.^[^ ^12^, ^13^, ^14^ ^]^ However, it is difficult for these wide bandgap semiconductors to absorb NIR light occupying ca. 50% of the solar spectrum to drive CO_2_ photoreduction reaction into hydrocarbon fuels. For this, different strategies involving the development of narrow bandgap semiconductor, vacancy engineering, doping engineering, and coupling plasmonic semiconductor, have been employed. Among these methods, plasmonic semiconductor has been confirmed to be the promising candidate to advance the NIR light driven photocatalytic CO_2_ reduction reaction because of unique optical absorption induced by localized surface plasmon resonance (LSPR) characteristic.^[^ ^2^, ^15^, ^16^, ^17^ ^]^ Therefore, improving the low hot electron transfer and separation efficiency of photogenerated carriers will be a breakthrough for developing effective plasmonic photocatalysts for CO_2_ photoreduction.

Compared with plasmonic metal/semiconductor Schottky junction, plasmonic metallic oxide photocatalysts can overcome the Schottky barrier at the metal‐semiconductor interface, further show high‐efficient hot electron transfer efficiency from plasmonic photocatalysts to CO_2_ molecules for protonation reaction of CO_2_.^[^ ^18^, ^19^, ^20^ ^]^ Moreover, to realize efficient electron‐hole separation, it is necessary to design defective/doped plasmonic metallic oxide photocatalysts, because the defect/doped sites may induce the generation of defect/doping energy level, which can reduce the bandgap of photocatalysts and act as trapping sites for facilitating carrier separation as well. Recently, molybdenum oxide‐based photocatalysts have been reported for photocatalytic CO_2_ reduction.^[^ ^11^, ^21^, ^22^, ^23^ ^]^ These studies mainly focused on improving light absorption by introducing LSPR induced by oxygen vacancies (OVs). The issues of the capacity of H_2_O activation for the supply of *H and the efficiency of hot electron transfer in photocatalytic CO_2_ reduction could not be resolved. Accordingly, developing defective molybdenum oxide‐based photocatalysts with NIR light responsive ability assists in boosting CO_2_ photoreduction activity, while fabricating doped photocatalysts with reduced bandgap and abundant dual‐metal sites is one way to tailor the hot electron transfer and carrier separation efficiency, and product selectivity of CO_2_ photoreduction in theory.

**Scheme 1:**
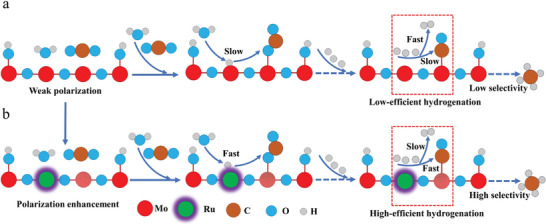
Substitution engineering to regulate CO_2_ photoreduction pathway and performance over a) MoO_3‐x_ and b) Ru‐substituted hydrogenated MoO_3‐x_ (Ru@H‐MoO_3‐x_) nanosheets. The Ru site was constructed by removal of a Mo atom, and Ru and Mo atoms are as the electron‐rich and electron‐deficient centers, respectively. In CO_2_ photoreduction process, electron‐rich Ru atom is as the H_2_O splitting sites, and electron‐deficient Mo as the CO_2_ reduction site.

Considering this, we rationally fabricated the polarization‐enhanced hydrogenated MoO_3‐x_ nanosheets by Ru substitution for selective CO_2_ photoreduction to CH_4_ under full spectrum and NIR light irradiation. Theoretical and experimental results indicate Ru atoms on the surface of the H‐MoO_3‐x_ matrix, achieving the charge redistribution of Ru and Mo atoms, which are as the electron‐rich and deficient centers for H_2_O splitting and CO_2_ reduction sites, respectively. Compared with weak polarization H‐MoO_3‐x_, the electron‐deficient Mo on Ru@H‐MoO_3‐x_ exhibits more favorable formation of *CHO in the process of *CO conversion due to the fast *H generation and transfer to *CO intermediates on electron‐rich Ru sites, and less energy is needed for further protonation to form CH_4_ with high selectivity (**Scheme** **1**). Formation of the OVs and Ru substitution intensifies the CO_2_ activation and the generation of *H with great efforts to achieve selective CO_2_ photoreduction into CH_4_. Furthermore, theoretical calculations suggest that Ru doping could narrow the bandgap and regulate the formation energy barrier of the key intermediates (*COOH and *CHO), thus making it a possible process for CO_2_ photoreduction to CH_4_. This study provides an opportunity for developing NIR light responsive photocatalyst for CO_2_ photoreduction into value‐added chemicals/fuels under sunlight illumination.

## 2 Results and Discussion

**Figure 1:**
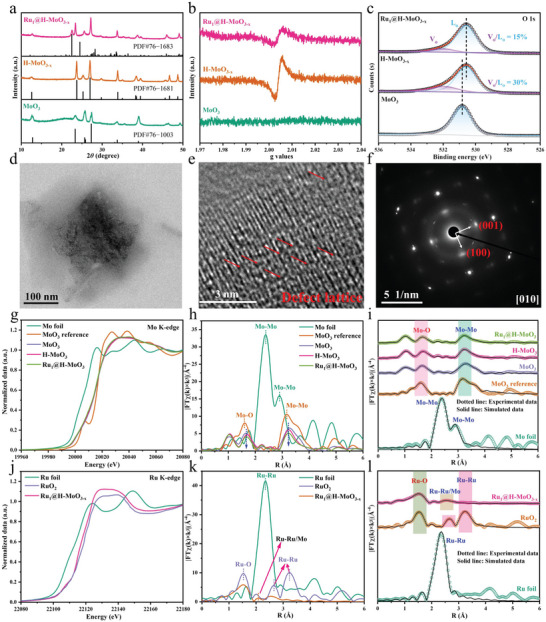
a) XRD patterns, b) ESR spectra, and c) O 1s XPS spectra of MoO_3_, H‐MoO_3‐x_, and Ru_1_@H‐MoO_3‐x_. d) TEM, e) HRTEM, f) SADE images of Ru_1_@H‐MoO_3‐x_ nanosheets. g) Normalized XANES spectra and h) EXAFS spectra in R‐space of the Mo K‐edge and i) corresponding Fourier transforms R space fitting results of Mo K‐edge. j) Normalized XANES spectra and k) EXAFS spectra in R‐space of the Ru K‐edge and l) corresponding Fourier transforms R space fitting results of Ru K‐edge.

The MoO_3_, hydrogenated MoO_3‐x_ (H‐MoO_3‐x_), and Ru substituted H‐MoO_3‐x_ (Ru_a_@H‐MoO_3‐x_; a represented the addition additive amount of RuCl_3_•nH_2_O and is equal to 0.5, 1, 2, and 4) nanosheets were synthesized via a hydrothermal/solvothermal approach. As illustrated in Figure S1 (Supporting Information), CH_3_CH_2_OH was specifically chosen to optimize the surface structure of MoO_3_ nanosheets, as it offers advantages over deoxygenation in the solvothermal process. The incorporation of H in the solvothermal process played a pivotal role, stabilizing the surface structure and electric neutrality resulting from the generation of surface Mo^5+^ species. As depicted in **Figure** **1**a and Figure S2 (Supporting Information), the XRD patterns of MoO_3_ synthesized using H_2_O as the solvent exhibited excellent agreement with α‐MoO_3_ crystal structure (JCPDS No. 76–1003), which has been further confirmed by Raman spectra (Figure S3, Supporting Information).^[^ ^24^, ^25^ ^]^ The phase structures of H‐MoO_3‐x_ and Ru@H‐MoO_3‐x_ synthesized using CH_3_CH_2_OH as the solvent are in line with α‐H_0.5_MoO_3_ crystal structure (JCPDS No. 76–1681).^[^ ^26^ ^]^ In addition, a new XRD characteristic peak appears at 2θ of 22.5°, corresponding to the (520) facet of α‐Mo_17_O_47_, and its intensity of the peak gradually amplifies with the increase of Ru doping content, indicating that Ru doping induced the formation of the quasi‐gold property MoO_3‐x_. The obvious EPR signal (g = 2.003) related to oxygen vacancies (OVs) could be observed in H‐MoO_3‐x_ and Ru_a_@H‐MoO_3‐x_ (Figure 1b). In general, the substitution of Mo^5+/6+^ by Ru^3+^ will reduce the concentration of OVs for ensuring electric neutrality of the catalysts, which can be demonstrated by ESR spectra (Figure S4 and Table S1, Supporting Information). The EPR signal intensity of Mo^5+^ species at the g‐value of 1.94 is found positively correlated with the concentration of OVs, which is negatively correlated with the doping amount of Ru, matching well with the XPS results. The above results provide strong proofs for the successful Ru substitution in H‐MoO_3‐x_ nanosheets. Moreover, Brunauer–Emmett–Teller (BET) surface areas of MoO_3_, H‐MoO_3‐x_, and Ru_1_@H‐MoO_3‐x_ were determined to be 16.03, 29.94, and 107.13 m^2^ g ^−1^, respectively (Figure S5, Supporting Information), indicating that the generation of OVs and Ru substitution dramatically expands the surface area. The high surface area implies that Ru_1_@H‐MoO_3‐x_ nanosheets could be a promising candidate as an adsorbent and catalyst for CO_2_ photoreduction.

The X‐ray photoelectron spectroscopy (XPS), X‐ray absorption near‐edge structure (XANES), and extended X‐ray absorption fine structure (EXAFS) analyses were carried out to explore electronic structure and local coordination environment of Ru and Mo atoms in Ru_1_@H‐MoO_3‐x_ nanosheets. As shown in Figure S6a (Supporting Information), the two peaks of Mo 3d spectra of MoO_3_, H‐MoO_3‐x_, and Ru_1_@H‐MoO_3‐x_ at 236.1 and 233.0 eV correspond to Mo^6+^.^[^ ^27^, ^28^, ^29^ ^]^ The other two peaks of H‐MoO_3‐x_, and Ru_1_@H‐MoO_3‐x_ centered at 234.7 and 231.6 eV are attributed to Mo^5+^. The peaks of Mo 3d spectrum of Ru_1_@H‐MoO_3‐x_ is negatively shifted by 0.1 eV compared with H‐MoO_3‐x_, suggesting the introduction of Ru species favors the electron enrichment of Mo atoms. Moreover, the fitted area ratio of Mo^5+^/Mo^6+^ of H‐MoO_3‐x_ and Ru_1_@H‐MoO_3‐x_ is decreased from 0.50 to 0.38, which is positive correlation with the concentration of OVs. The O 1s peaks at 531.9 and 530.5 eV are assigned to OVs (V_o_) and lattice oxygen (L_o_), respectively (Figure 1c).^[^ ^28^, ^30^ ^]^ The ratios of V_o_/L_o_ of H‐MoO_3‐x_ and Ru_1_@H‐MoO_3‐x_ are 30% and 15%. The above results confirmed the low‐cost state Ru substitution in H‐MoO_3‐x_ nanosheets reduces the concentration of OVs, which is consistent with the EPR spectra.^[^ ^31^ ^]^ In the C 1s + Ru 3d spectrum of Ru_1_@H‐MoO_3‐x_, the peaks at 285.6 and 281.6 eV are assigned to Ru 3d (Figure S6b, Supporting Information), suggesting the successful introduction of Ru species in H‐MoO_3‐x_ structure.^[^ ^27^ ^]^ The similar results can also be observed in Ru_a_@H‐MoO_3‐x_ (a = 0.5, 2, and 4) samples (Figure S7, Supporting Information).

Scanning electron microscopy (SEM) (Figure S8, Supporting Information) and transmission electron microscopy (TEM) images (Figures S9 and S10, Supporting Information; Figure 1d) reveal the ultrathin sheet‐like morphology of MoO_3_, H‐MoO_3‐x_, and Ru_1_@H‐MoO_3‐x_ products, potentially suggesting the atomic‐scale size of the Ru embedded in the H‐MoO_3‐x_ matrix. According to the high‐resolution TEM (HRTEM) image of Ru_1_@H‐MoO_3‐x_ (Figure 1e), the missing atoms can be distinguished in lattice fringes, further suggesting the formation of OVs. The angle of adjacent spots of Ru_1_@H‐MoO_3‐x_ labeled in the SAED is consistent with the theoretical angle between the (001) and (100) planes (Figure 1f), demonstrating that Ru_1_@H‐MoO_3‐x_ nanosheets are grown along the [010] zone axis.^[^ ^32^ ^]^ Moreover, the energy dispersion spectrum (EDS) elemental mapping (Figure S11, Supporting Information) clearly shows the homogeneous distribution of Ru, Mo, and O elements, which further verifies that Ru atoms have been successfully embedded into the matrix of Ru_1_@ H‐MoO_3‐x_ nanosheets.

The X‐ray absorption near‐edge structure (XANES) of the Mo K‐edge is shown in Figure 1g. The energy absorption edge and the height of Ru_1_@H‐MoO_3‐x_ are closed to MoO_3_ and H‐MoO_3‐x_, and lower than commercial MoO_3_ reference, proving the existence of low valence‐state Mo^δ+^ (δ < 6).^[^ ^33^ ^]^ For Ru_1_@H‐MoO_3‐x_, the peaks at 1.67 and 3.23 Å in the R space correspond to Mo‐O and Mo‐Mo paths and the peaks of H‐MoO_3‐x_ and Ru_1_@H‐MoO_3‐x_ shift to low energy compared with MoO_3_, indicating slight variations in Mo‐O and Mo‐Mo bond length (Figure 1h), further evidenced by the wavelet transform (WT) plots of the Mo K‐edge of Ru_1_@H‐MoO_3‐x_ (Figure S12, Supporting Information). The coordination structures of Mo were further analyzed by fitting the k3‐weighted FT‐EXAFS spectra (Figure 1i), and the fitted parameters are summarized in Table S2 (Supporting Information). Ru_1_@H‐MoO_3‐x_ showed Mo‐O and Mo‐Mo coordination structures with coordination numbers of 4.3 (Mo‐Mo) and 4.7 (Mo‐O), respectively. The Ru_1_@H‐MoO_3‐x_ with decreased concentration of OVs, which reveals lower Mo‐O and Mo‐Mo coordination numbers compared with H‐MoO_3‐x_, suggesting the Ru successfully replaces Mo atoms in Ru_1_@H‐MoO_3‐x_ structure. The formation of Ru‐O coordination structures further verified the above results (Figure 1j–l). The adsorption energy of Ru K‐edge of Ru_1_@H‐MoO_3‐x_ is lower than RuO_2_ and higher than Ru foil reference, revealing that the valence‐state of Ru in the Ru_1_@H‐MoO_3‐x_ is between 0 and 4 (Figure 1j).^[^ ^34^ ^]^ The Ru K‐edge EXAFS and curve fitting result of Ru_1_@H‐MoO_3‐x_ show that peaks at 2.07 Å were observed and assigned to Ru‐Ru/Mo scattering path (first coordination shell). This feature was shorter than the first Ru‐Ru coordination shell feature in RuO_2_ foil (2.01 Å) (Figure 1k). The scattering path of Ru‐Ru/Mo and Ru‐O in Ru_1_@H‐MoO_3‐x_ with coordination numbers are ≈2.6 and 5.1 (Figure 1l). The wavelet transform (WT) plots for the Ru K‐edge further evidence the existence of the Ru‐Ru/Mo scattering path in Ru_1_@H‐MoO_3‐x_ (Figure S13, Supporting Information). Therefore, the above results, combining the characterization of XPS and EDS‐mapping, strongly confirm that the Ru atom substitution in Ru_1_@H‐MoO_3‐x_ with the Ru‐Mo and Ru‐O coordination and not the Ru metal clusters.

**Figure 2:**
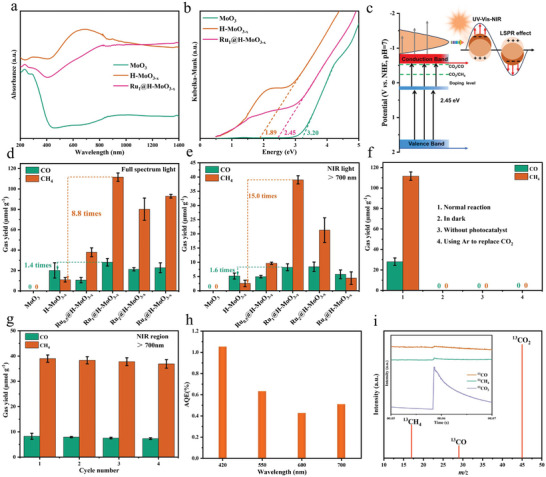
a) UV–vis‐NIR diffuse reflectance spectra and b) the corresponding optical bandgaps of MoO_3_, H‐MoO_3‐x_, and Ru_1_@H‐MoO_3‐x_. c) Schematic illustration of LSPR effect on Ru_1_@H‐MoO_3‐x_. Photocatalytic CO_2_ reduction product evolution as a function of light irradiation times on the as‐prepared samples under d) full spectrum light and e) NIR light irradiation. f) Photocatalytic CO_2_ reduction activity for Ru_1_@H‐MoO_3‐x_ under different experimental conditions. g) Cycling tests of photocatalytic CO_2_ reduction under NIR light irradiation over Ru_1_@H‐MoO_3‐x_. h) Apparent quantum efficiency (AQE) for CO_2_ photoreduction over Ru_1_@H‐MoO_3‐x_. i) MS spectrum to detect CO_2_ photoreduction products using ^13^CO_2_ as the CO_2_ source.

**Figure** **2**a depicts the optical property of MoO_3_, H‐MoO_3‐x_, and Ru_1_@H‐MoO_3‐x_. The H‐MoO_3‐x_ and Ru_1_@H‐MoO_3‐x_ samples after introducing OVs show a strong absorption from ranging 200–1400 nm compared with MoO_3_. The spectrum light adsorption property is associated with the localized surface plasmon resonance (LSPR) of Mo^6+^/Mo^5+^ in the samples, ^[^ ^35^, ^36^ ^]^ resulting from the free electrons and/or the deficient oxygen‐induced small polarons. The carrier density (*N_c_*) for the samples can be determined by the following Equation (1).^[^ ^37^ ^]^

(1) $$\;N_{c}=2e_{0}^{-1}\varepsilon^{-1}\varepsilon_{0}^{-1}{\left|d\;\left(C^{-2}/dV\right)\right|}^{-1}$$

where e_0_, ε_0_, and ε represent the electron charge, vacuum permittivity, and the dielectric constant, respectively. As shown in Figure S14 (Supporting Information), Ru_1_@H‐MoO_3‐x_ exhibits a much lower slope compared with MoO_3_ and H‐MoO_3‐x_ based on Equation (1), suggesting Ru_1_@H‐MoO_3‐x_ has a higher value of *N_c_*. Accordingly, the existence of OVs causes the increasement of excess free electrons in Ru_1_@H‐MoO_3‐x_, which results in the LSPR effect for the sample. The similar LSPR induced by OVs can be observed on Bi_2_O_3‐x_ and MoO_2_.^[^ ^38^, ^39^ ^]^ The bandgaps of MoO_3_, H‐MoO_3‐x_, and Ru_1_@H‐MoO_3‐x_ are calculated to be 3.20, 1.89, and 2.45 eV (Figure 2b), suggesting OVs can efficiently decrease the bandgap of MoO_3_. However, the bandgaps of Ru_1_@H‐MoO_3‐x_ is larger than that of H‐MoO_3‐x_, which may originate from the decreased concentration of OVs induced by Ru substitution. Mott‐Schottky plots show that all the samples display a positive slope, showing their n‐type semiconductor property (Figure S14, Supporting Information).^[^ ^40^ ^]^ According to the flat potentials and their bandgap, the band structure of MoO_3_, H‐MoO_3‐x_, and Ru_1_@H‐MoO_3‐x_ are depicted in Figure S15 (Supporting Information). Therefore, a possible band structure and LSPR mechanism of Ru_1_@H‐MoO_3‐x_ is presented in Figure 2c.

The photocatalytic activities of the target products with full spectrum and NIR light irradiation were evaluated. As shown in Figure 2d, CO and CH_4_ are the main products of CO_2_ photoreduction for H‐MoO_3‐x_ and a series of Ru@H‐MoO_3‐x_, and no products were detected for MoO_3_, which is attributed to its lower conduction band (CB) potential than CO_2_ photoreduction for the generation of products. Among these photocatalysts, Ru_1_@H‐MoO_3‐x_ reveals the best CO_2_ photoreduction activity under full spectrum and NIR light irradiation. The yields of CO and CH_4_ of Ru_1_@H‐MoO_3‐x_ are 28.0 and 111.6 µmol g_catalyst_^−1^, respectively, which is 1.4 and 8.8 times than H‐MoO_3‐x_ under full spectrum light irradiation. More importantly, under NIR light irradiation, Ru_1_@H‐MoO_3‐x_ still showed superior CO_2_ photoreduction activity and the yields of CO and CH_4_ are 8.2 and 39.0 µmol g_catalyst_^−1^, which was 1.6 and 15.0 times much higher than that of H‐MoO_3‐x_ (Figure 2e). The CH_4_ yield rate is superior to most of the reported works (Table S3, Supporting Information). It is worth noting that small amount of H_2_ is also be detected with full spectrum light, generated by the reduction of *H. With the introduction of Ru species, the yield of H_2_ is decreased and simultaneously the contact angle of H_2_O increases, suggesting the *H reduction reaction can be inhibited. This result favors the supply of *H for CO_2_ reduction reaction (Figures S16 and S17, Supporting Information). The product selectivity of CH_4_ is up to ≈90% under full spectrum or NIR irradiation (Figure S18, Supporting Information). The turnover number (TON) is also calculated (Figure S19, Supporting Information). The above results indicate that Ru_1_@H‐MoO_3‐x_ exhibits superior CO_2_ photoreduction performance for CH_4_ generation with high selectivity. The control experiments were performed to explore the source of products of photocatalytic CO_2_ reduction (Figure 2f). No reduction products were detected in the dark, in the absence of photocatalyst, or in Ar atmosphere, indicating that the products result from the photoreaction of CO_2_ in photocatalytic process. As shown in Figure 2g, there is no obvious decline after four cycles. The apparent quantum efficiency (AQE) for CO_2_ photoreduction over Ru_1_@H‐MoO_3‐x_ can be up to 0.51% under NIR light (700 nm) irradiation (Figure 2h). The AQE at 700 nm with the optical power to 0.70 W was also measured, which is apparently higher than of 0.40 W (Figures S20 and S21, Supporting Information). The XRD and XPS further confirm the stability of Ru_1_@H‐MoO_3‐x_ in CO_2_ photoreduction reaction (Figure S22, Supporting Information). In addition, ^13^C isotope tracer experiments were performed to trace the carbon source of the reduction products (Figure 2i). When the carbon source is ^13^CO_2_, the ^13^CO and ^13^CH_4_ are detected, indicating that the carbon source of products is indeed from CO_2_ photoreduction. Temperature‐time variation curves were tested to explore the heating rate of the sample under full‐spectrum irradiation (Figure S23, Supporting Information). The core temperature of Ru_1_@H‐MoO_3‐x_ in the reaction vessel rapidly increases to 96 °C in 150 s with full spectrum irradiation, which is higher than H‐MoO_3‐x_. The heat generated by photothermal effect is beneficial to the CO_2_ photoreduction reaction in dynamics.

**Figure 3:**
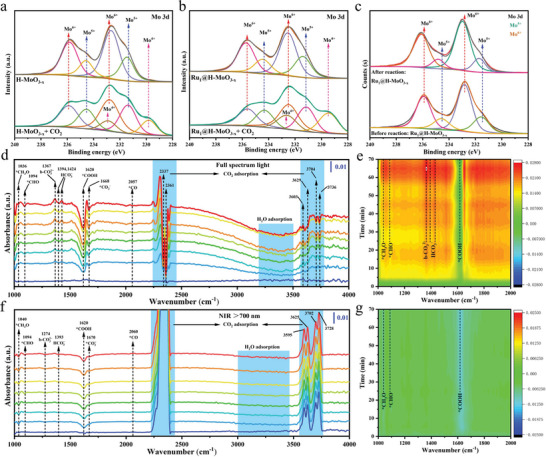
NAP‐XPS Mo 3d spectra of a) H‐MoO_3‐x_ and b) Ru_1_@H‐MoO_3‐x_, when introducing 1 mbar of Ar and Ar + CO_2_ at room temperature, respectively. c) XPS spectra of Ru_1_@H‐MoO_3‐x_ before and after CO_2_ photoreduction. In situ FTIR spectra of Ru_1_@H‐MoO_3‐x_ under d) full spectrum and f) NIR light irradiation and the corresponding 2D contour color fill plots in the wavenumber range from 1000 to 2000 cm^−1^ (e,g), respectively.

To study CO_2_ adsorption on Ru_1_@H‐MoO_3‐x_, CO_2_ was introduced to Ru_1_@H‐MoO_3‐x_ surface without light illumination. The adsorption sites on Ru_1_@H‐MoO_3‐x_ for CO_2_ were then investigated by near ambient‐pressure (NAP) XPS. **Figures** **3**a,b reveal that before and after CO_2_ are introduced into the surface of H‐MoO_3‐x_ and Ru_1_@H‐MoO_3‐x_. For H‐MoO_3‐x_, the Mo^6+^ shifts to higher binding energy side and Mo^5+^ behaves the opposite direction. More importantly, a new valence state of Mo^4+^ appears in CO_2_ atmosphere.^[^ ^26^ ^]^ Additionally, the variation trends of Mo^6+^ and Mo^5+^ present the opposite path compared with Ru_1_@H‐MoO_3‐x_ and Mo^4+^ is also observed. The above results suggest strong interaction between the outermost valence electrons of Mo with CO_2_. The generation of Mo^4+^ confirms the CO_2_ is activated by electrical discharge and Mo^4+^ is the key species of CO_2_ adsorption and activation. The Mo 3d XPS spectra before and after used Ru_1_@H‐MoO_3‐x_ are also analyzed. The peaks of Mo^6+^ and Mo^5+^ are detected, but the Mo^4+^ cannot be observed resulting from the unstable property in the presence of O_2_ (Figure 3c). In situ Fourier transform infrared spectroscopy (FTIR) was carried out to observe the reaction intermediates and explore the relative mechanism of CO_2_ photoreduction process. The new infrared peak at 1668 (Figure 3d) and 1670 cm^−1^ (Figure 3f) for Ru_1_@H‐MoO_3‐x_ under full spectrum and NIR light irradiation, respectively, were detected, and they belong to *CO_2_^−^, which is the key intermediate of CO_2_ adsorption and activation.^[^ ^41^ ^]^ The peaks at 1620 cm^−1^ in Figure 3d–g correspond to the *COOH groups, which is considered as the crucial intermediate during CO_2_ photoreduction to CO/CH_4_.^[^ ^9^ ^]^ The absorption at 1036 cm^−1^ in Figure 3d,e and at 1040 cm^−1^ in Figure 3f,g are attributed to the *CH_3_O group, while the peak at 1094 cm^−1^ in Figure 3d–g are ascribed to the *CHO group, and both the *CHO and *CH_3_O groups are the crucial intermediate products of photocatalytic CO_2_ reduction into CH_4_.^[^ ^42^ ^]^ The peaks at 1367 cm^−1^ in Figure 3d and at 1247 cm^−1^ in Figure 3f could be assigned to symmetric stretching of b‐CO_3_^2−^ groups. The absorption bands at 1394 and 1424 cm^−1^ in Figure 3d,e and at 1393 cm^−1^ in Figure 3f are regarded as the *HCO_3_ species. More importantly, CO* absorption bands at 2057 cm^−1^ in Figure 3d and at 2060 cm^−1^ in Figure 3f present a minuscule peak, suggesting the generation of *CO and the *CO intermediates on the Ru_1_@H‐MoO_3‐x_ surface may be rapidly protonated to produce CHO* rather than being desorbed from the catalyst surface to form CO molecules, thus resulting in the high selectivity of CH_4_ generation in photocatalytic CO_2_ reduction reaction.^[^ ^9^, ^43^ ^]^ The peaks from 2200–4000 cm^−1^ (Figures 3d,f) are mainly identified as the CO_2_ and H_2_O adsorption.

**Figure 4:**
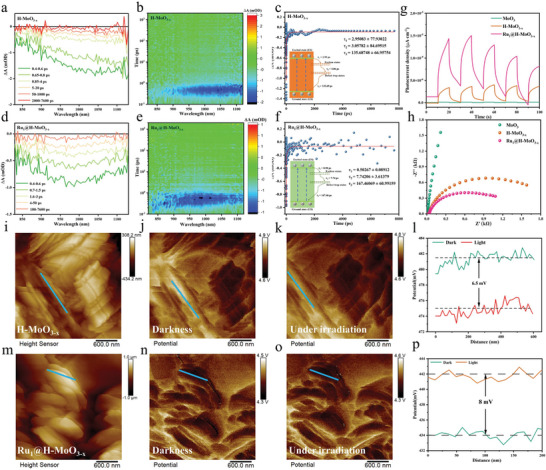
TA spectra, corresponding 3D contour plots, and TA kinetics traces of a–c) H‐MoO_3‐x_ and d–f) Ru_1_@H‐MoO_3‐x_, respectively. g) Transient photocurrent response h) EIS spectra, and PL spectra of H‐MoO_3‐x_ and Ru_1_@H‐MoO_3‐x_. AFM height images, KPFM images, and surface potential analysis (from left to right) of i–l) H‐MoO_3‐x_ and m–p) Ru_1_@H‐MoO_3‐x_ in the dark and under light irradiation. SPV change by subtracting the potential under dark conditions from that under irradiation (∆CPD = CPD_dark_‐ CPD_light_).

To gain deeper insights into the kinetics process of photogenerated carriers, the femtosecond transient absorption (pump‐probe) (fs‐TA) spectroscopy was performed to obtain the carriers’ dissociation process and lifetime scale.^[^ ^44^, ^45^ ^]^ The fs‐TA spectra of H‐MoO_3‐x_ (**Figures** **4**a,b) and Ru_1_@H‐MoO_3‐x_ (Figures 4d,e) exhibited continuous negative absorption in the wavelength range from 800 to 1200 nm corresponding to the ground‐state bleach (GSB) signal. Figures 4c,f show the fs‐TA kinetic profiles at 950 nm of H‐MoO_3‐x_ and Ru_1_@H‐MoO_3‐x_, and their signal can be well‐fitted with triexponential results. The fs‐TA spectra of H‐MoO_3‐x_ and Ru_1_@H‐MoO_3‐x_ were fitted by a tri‐exponential function and the fitting parameters are τ_1_ = 2.95 ps, τ_2_ = 3.05 ps, τ_3_ = 135.69 ps for H‐MoO_3‐x_, and τ_1_ = 0.50 ps, τ_2_ = 7.74 ps, τ_3_ = 167.46 ps for Ru_1_@H‐MoO_3‐x_. Photogenerated electron relaxation in the CB of H‐MoO_3‐x_ and Ru_1_@H‐MoO_3‐x_ occurred through three pathways: exciton‐mediated states (τ_1_), defect trap states (τ_2_), and recombination of e^−^ and h^+^(τ_3_). Notably, the lifetime components of Ru_1_@H‐MoO_3‐x_ (τ_1_ = 0.50 ps) are much shorter than that of H‐MoO_3‐x_ (τ_1_ = 2.95 ps), showing the faster transfer to exciton‐mediated states. The longer lifetime of τ_2_ (7.74 ps) for Ru_1_@H‐MoO_3‐x_ compared with H‐MoO_3‐x_ favor to the transfer of photogenerated e^−^ to defect trap states, further prolong the lifetime of τ_3_ for retarding the recombination process of photogenerated e^−^ and h^+^. The efficient separation of photogenerated carriers further supported by transient photocurrent (Figure 4g), electrochemical impedance spectroscopy (EIS) (Figure 4h), and transient photoluminescence (PL) (Figure S24, Supporting Information) measurements. Kelvin‐probe force microscopy (KPFM) was also performed to clarify the separation and transport mechanism of photogenerated carriers. More negative surface photovoltage (SPV) signal change before and after light irradiation represents a much higher concentration of photoinduced electrons. The H‐MoO_3‐x_ (Figures 4i–l) and Ru_1_@H‐MoO_3‐x_ (Figures 4m–p) reveal 6.5 and 8.0 mV negative change before and after light irradiation, corresponding to band‐to‐band transition.^[^ ^46^, ^47^ ^]^ More negative SPV signal change was observed on Ru_1_@H‐MoO_3‐x_ than H‐MoO_3‐x_, confirming that Ru_1_@H‐MoO_3‐x_ enables to facilitate the electron‐hole separation to realize high concentration accumulation of photoelectrons on its surface, which supports CO_2_ photoreduction process.

**Figure 5:**
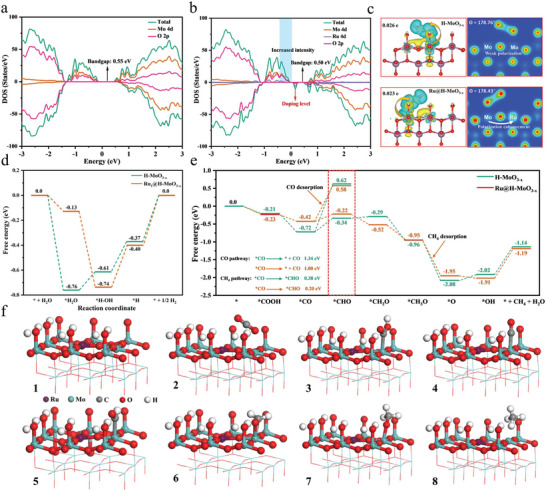
Calculated DOS of a) H‐MoO_3‐x_ and b) Ru@H‐MoO_3‐x_. c) Calculated models and electron localization function results of H‐MoO_3‐x_ and Ru@H‐MoO_3‐x_ to CO_2_. d) Free energy diagram for water splitting with the generation of *H, and e) CO_2_ photoreduction to CH_4_ over H‐MoO_3‐x_ and Ru_1_@H‐MoO_3‐x_. f) The corresponding structure models for CO_2_ photoreduction to CH_4_ from 1 to 8.

Density functional theory (DFT) calculations are employed to investigate the function of Ru substitution on the electric structure. **Figures** **5**a,b clearly display that the presence of Ru substitution results in the generation of a new doping level, which makes the bandgap to decrease from 0.55 to 0.50 eV. Moreover, the Ru@H‐MoO_3‐x_ reveals increased DOS intensity in comparison with the H‐MoO_3‐x_, suggesting its higher hole concentration at the VB edge. The above results indicate the higher transition probability of photogenerated electrons to the CB of Ru@H‐MoO_3‐x_ under solar light irradiation for improving the CO_2_ photoreduction performance. The CO_2_ molecule adsorbed on the surface of Ru_1_@H‐MoO_3‐x_ displayed larger bond angle change compared with H‐MoO_3‐x_, thus highlighting the higher thermodynamic feasibility for C═O bond dissociation (Figure 5c). The Bader charge results show that 0.023 and 0.026 electrons of CO_2_ transfer to Ru@H‐MoO_3‐x_, and H‐MoO_3‐x_ during CO_2_ adsorption and activation, evidencing the chemical adsorption of CO_2_ on the surface of Ru@H‐MoO_3‐x_, and H‐MoO_3‐x_. Electron localization function results manifest the electrons tend to transfer from Mo to Ru, and Ru and Mo become the electron‐rich and deficient center after Ru substitution. This phenomenon enhances the charge internal polarization, which is beneficial to the separation of photogenerated carriers. By comparing the PDOS of CO_2_ in gas phase, H‐MoO_3‐x_ and Ru_1_@H‐MoO_3‐x_, it can be found that the PDOS orbital of CO_2_ adsorbed state is lower than that of gas phase. The anti‐bonding orbital above 0 of Ru_1_@H‐MoO_3‐x_ is higher than that of H‐MoO_3‐x_ and the electron occupation states are more locally concentrated and distributed, indicating the adsorption CO_2_ on the surface of Ru_1_@H‐MoO_3‐x_ is stronger than H‐MoO_3‐x_ (Figure S25, Supporting Information). The CO_2_ adsorption measurements were performed to verify enhanced CO_2_ adsorption over Ru_1_@H‐MoO_3‐x_ (Figure S26, Supporting Information).

To gain an in‐depth understanding of CO_2_ photoreduction process, the free energies of the possible reaction paths involved on H‐MoO_3‐x_ and Ru@H‐MoO_3‐x_ in H_2_O splitting and photocatalytic CO_2_ reduction reaction process were calculated (Figures 5d,e). As shown in Figure 5d, the adsorption of H_2_O proceeds spontaneously and tends to adsorb on the surface of H‐MoO_3‐x_ and Ru@H‐MoO_3‐x_ surface. The energy barrier of *H‐OH generation on the H‐MoO_3‐x_ surface is 0.15 eV, while on Ru@H‐MoO_3‐x_ surface is exothermic reaction, suggesting it can proceed spontaneously. The generation of *H, which is the key species of CO_2_ reduction on the Ru@H‐MoO_3‐x_ surface. Moreover, the secondary reaction of H_2_ generation can be suppressed due to the higher energy barrier on the Ru@H‐MoO_3‐x_ surface (∆E = 0.40 eV) than that of H‐MoO_3‐x_ surface (∆E = 0.37 eV), which is favorable to the generation of ∗H on the Ru@H‐MoO_3‐x_ surface for hydrogenation reaction of CO_2_. Figures 5e,f show the Gibbs free energy pathways of CO_2_ reduction to CH_4_ for H‐MoO_3‐x_ and Ru@H‐MoO_3‐x_. After introducing Ru, the Gibbs free energy of *COOH formation for Ru@H‐MoO_3‐x_ is less than 0 (−0.21 eV), and that of H‐MoO_3‐x_ is −0.23 eV. This suggests CO_2_ hydrogenation is easier and more spontaneous with the substitution of Ru. The formation energy of *CO protonation to *CHO for H‐MoO_3‐x_ and Ru@H‐MoO_3‐x_ is 1.34 and 1.00 eV, which is the largest energy barrier of all the basic steps that be considered as the rate‐determining step. After introducing Ru into H‐MoO_3‐x_, the corresponding rate‐determining step energy barrier decreases to 1.00 eV, indicating that Ru@H‐MoO_3‐x_ is more likely to drive photocatalytic CO_2_ reduction reaction. Meanwhile, the energy barrier *CO desorption to CO gas on both H‐MoO_3‐x_ and Ru@H‐MoO_3‐x_ is higher than that of *CO protonation, suggesting *CO protonation is easier than CO desorption. Moreover, the energy barrier of *CHO (0.38 eV) on Ru@H‐MoO_3‐x_ is lower than that on H‐MoO_3‐x_ (0.20 eV), testifying Ru‐Mo dual sites are more propitious to the generation of *CHO groups with the more supply of *H compared with Mo‐Mo sites, ensuring the high selectivity generation of CH_4_. Both experimentally and theoretically, Ru@H‐MoO_3‐x_ exhibits a better photocatalytic CO_2_ reduction performance into CO or CH_4_ than H‐MoO_3‐x_.

## 3 Conclusion

In summary, we have fabricated functional Ru‐Mo dual metal sites in plasmonic Ru@H‐MoO_3‐x_ nanosheet photocatalyst with electron‐rich and deficient centers for regulating the selectivity and reactivity of CO_2_ photoreduction. Experimental and theoretical calculation results verify that the Ru substitution creates an enhanced local charge accumulation on the Ru@H‐MoO_3‐x_ nanosheets, and the electron‐rich center Ru atom and electron‐deficient center Mo atom can act as the site for H_2_O and CO_2_ adsorption and activation. Moreover, the synergistic effect of Ru substitution and OVs inhibits the recombination of photogenerated carriers, simultaneously facilitates the CO_2_ adsorption and activation as well as the supply of *H, respectively. Compared with weak polarization H‐MoO_3‐x_, the enhanced polarization Ru@H‐MoO_3‐x_ exhibits more favorable formation of *CHO in the process of *CO conversion, and less energy is needed for further protonation to form CH_4_. The optimized Ru@H‐MoO_3‐x_ nanosheets show enhanced photocatalytic CO_2_ reduction activity into CH_4_ under NIR light irradiation and the product selectivity of CH_4_ reaches ≈96%. This work provides a feasible strategy for the design of dual functional active centers for selective CO_2_ photoreduction into solar fuels.

## Conflict of Interest

The authors declare no conflict of interest.

## Supporting information

Supporting Information

## Data Availability

The data that support the findings of this study are available from the corresponding author upon reasonable request.
