# Supplementary material for: Selective CO2 Photoreduction into CH4 Triggered by the Synergy between Oxygen Vacancy and Ru Substitution under Near‐Infrared Light Irradiation
Source: Adv Sci (Weinh). 2024 Jul 9;11(34):2405668. doi: 10.1002/advs.202405668 (PMC11425646; doi:10.1002/advs.202405668)
Supplement: Supplementary file 1 — Supporting Information [file ADVS-11-2405668-s001.docx]

Supporting Information

**Selective CO_2_ Photoreduction into CH_4_ Triggered by the Synergy between Oxygen Vacancy and Ru Substitution under Near-Infrared Light Irradiation**

*Jun Li, Xinglong Liu, Xi Wu, Zhongyi Liu*, Zaiwang Zhao, Yifeng Liu, Shixue Dou, and Yao Xiao**

J. Li, X. L. Liu, X. Wu, Z. Y. Liu

Henan Institute of Advanced Technology, College of Chemistry, Zhengzhou University, Zhengzhou 450052, China.

E-mail: liuzhongyi@zzu.edu.cn

Y. F. Liu, Y. Xiao
College of Chemistry and Materials Engineering, Wenzhou University, Wenzhou, 325035, P.R. China.

E-mail: xiaoyao@wzu.edu.cn

S. X. Dou
Institute of Energy Materials Science (IEMS), University of Shanghai for Science and Technology, Shanghai 200093, PR China.

Z. W. Zhao
College of Energy Materials and Chemistry, College of Chemistry and Chemical Engineering, Inner Mongolia University, Hohhot 010070, P. R. China

1. **Experimental section**

***1.1. Synthesis of MoO_3_***

In a typical synthesis, Mo powder (192mg) and deionized water (24 mL) were added to a polyphenylene (PPL) liner (50 mL) and stirred. Then, 3 mL H_2_O_2_ (30 wt%) was slowly added into the above suspension. Subsequently, PPL liner was sealed in a stainless-steel autoclave, heated at 160 °C for 12 h in an oven, and naturally cooled to room temperature. The resulting products were washed with deionized water and ethanol, and dried at 60 °C in a vacuum oven overnight.

***1.2. Synthesis of H-MoO_3-x_***

In a typical synthesis, Mo powder (192mg) and anhydrous ethanol (24 mL) were added to a polyphenylene (PPL) liner (50 mL) and stirred. Then, 3 mL H_2_O_2_ (30 wt%) was slowly added into the above suspension. Subsequently, PPL liner was sealed in a stainless-steel autoclave, heated at 160 °C for 12 h in an oven overnight, and naturally cooled to room temperature. The resulting products were washed with deionized water and ethanol, and dried at 60 °C in a vacuum overnight.

***1.3. Synthesis of Ru@H-MoO_3-x_***

In a typical synthesis, Mo powder (192mg) and anhydrous ethanol (24 mL) were added to a polyphenylene (PPL) liner (50 mL) and stirred. Then, 3 mL H_2_O_2_ (30 wt%) was slowly added into the above suspension. Subsequently, a certain amount of RuCl_3_ was added into the above suspensions and stirred continuously for 30 min. Finally, PPL liner was sealed in a stainless-steel autoclave, heated at 160 °C for 12 h in an oven, and naturally cooled to room temperature. The resulting products were washed with deionized water and ethanol, and dried at 60 °C in a vacuum overnight. When the dosage of RuCl_3_ aqueous solution (20 mg/mL) were 50, 100, 200 and 400 μL, the obtained Ru@H-MoO_3-x_ were labeled as Ru_0.5_@H-MoO_3-x_, Ru_1_@H-MoO_3-x_, Ru_2_@H-MoO_3-x_, and Ru_4_@H-MoO_3-x_, respectively.

***1.4. Characterization***

The crystal structures of the as-prepared samples were detected by X-ray diffraction (XRD, Panalytical XPert Pro X-ray diffractometer). Transmission electron microscope (TEM) (FEI Tecnai F20) was conducted to observe the morphologies of the samples. Chemical compositions and valence band information of the samples were analyzed using X-ray photoelectron spectroscopy (XPS) (Thermo Scientific K-Alpha). Electron paramagnetic resonance (EPR) was measured on a Bruker BioSpin EMX-9.5/12. UV-vis-NIR diffuse reflectance spectra (DRS) were recorded on a Shimadzu UV-3600 spectrophotometer. Micromeritics ASAP 2020 automatic gas adsorption system was used to value the Brunauer-Emmett-Teller (BET) surface area and pore size distribution. Carrier dynamics were probed by transient absorption spectroscopy (TAS, Helios, ultrafast systems). CO/CO_2_-TPD experiments were performed on a Micromeritics Auto Chem 2950HP. The CO_2_ adsorption isotherms were acquired on a Micromeritics ASAP 2460.

***1.5. Electrochemical and photoelectrochemical measurements***

Transient photocurrent response, electrochemical impedance and Mott-Schottky measurements were conducted in a three-electrode quartz cell on a CHI660E electrochemical workstation (CHI, China), and 0.5 M Na_2_SO_4_ was used as the electrolyte. Ag/AgCl electrode, Pt wire and photocatalyst-coated fluorine-doped tin oxide (FTO 1.5 cm × 2.2 cm) are used as reference, counter electrode, and working electrode, respectively. The prepared method of working electrode is as follows: the sonication-dispersed suspensions of 10 mg of photocatalyst, 1 ml of ethanol, and 40 μL of Nafion solution were pipetted onto FTO with 400 μL, and then dried under the thermal radiation of NIR light. A 300 W Xenon lamp (PLS-SXE300, Perfectlight, China) was selected as the light source.

***1.6. In situ Fourier transform infrared spectroscopy (FTIR) analysis***

*In situ* FTIR experiments were performed on a Bruker Vertex 70 Fourier Transform Infrared Spectrometer. The photocatalyst was evenly dispersed onto an *in situ* cell. Subsequently, a mixed gas of CO_2_ and H_2_O was introduced into the *in situ* cell, and the adsorption data was recorded every 4 min. Finally, the data was recorded at the same time interval under full spectrum or NIR light irradiation.

***1.7. Computational method***

The Density Functional Theory (DFT) calculations were performed by Vienna *Ab-initio* Simulation Package (VASP)^1^ with the projector augmented wave (PAW) method^2^. The exchange-functional was treated using the Perdew-Burke-Ernzerhof (PBE)^3^ functional, in combination with the DFT-D3 correction^4^. The slab models of H-MoO_3-x_ and Ru@H-MoO_3-x_ by introducing oxygen vacancies in each system were built. A cut-off energy of the plane-wave basis was set at 450 eV. For the optimization of both geometry and lattice size, the Brillouin zone integration was set with a Monkhorst-Pack *k*-point mesh of 3×3×1. The self-consistent calculations applied a convergence energy threshold of 10^-5^ eV. The equilibrium geometries and lattice constants were optimized with maximum stress on each atom within 0.02 eV Å^-1^. Spin polarization method was used to describe magnetism of the models.

***1.8. Photocatalytic CO_2_ reduction***

20 mg of photocatalyst was dispersed in 5 mL deionized H_2_O with continuously ultrasonication to obtain a uniform suspension. The above suspension was then dropped on a quartz disc with a diameter of 5 cm and dried at 70 °C to obtain the catalyst-coated quartz disc. Afterwards, the quartz disc was placed in a custom-made reaction vessel (250 mL) with 5 mL deionized H_2_O on the bottom. High-purity CO_2_ (99.999%) was used as the carbon source. A 300 W Xenon lamp (PLS-SXE300, Perfectlight, China) was used as light source. The gaseous products were recorded by a gas chromatography (GC) 7820 (Zhongkehuifen China).

**2. Figure Captions**

**Figure S1.** Synthetic diagram of MoO_3_, H-MoO_3-x_, and Ru@H-MoO_3-x_ nanosheets.

**Figure S2.** XRD patterns of a serious of Ru@H-MoO_3-x_ nanosheets.

**Figure S3.** Raman spectra of MoO_3_, H-MoO_3-x_, and Ru@H-MoO_3-x_.

**Figure S4.** ESR spectra of Ru@H-MoO_3-x_ nanosheets.

**Figure S5.** N_2_ adsorption/desorption isotherms of MoO_3_, H-MoO_3-x_, and Ru@H-MoO_3-x_ nanosheets.

**Figure S6.** XPS spectra of MoO_3_, H-MoO_3-x_, and Ru_1_@H-MoO_3-x_: (a) Mo 3d, (b) Ru 3d.

**Figure S7.** XPS spectra for a series of Ru@H-MoO_3-x_: (a) Mo 3d, (b) O 1s, (c) Ru 3d and C 1s.

**Figure S8.** SEM images of (a-c) MoO_3_, (d-f) H-MoO_3,_ and (g-i) Ru_1_@H-MoO_3_ nanosheets.

**Figure S9.** (a) TEM, (b) HRTEM, (c) SADE, and (d-f) element mapping of Mo and O of MoO_3_ nanosheets.

**Figure S10.** (a-b) TEM, (c) HRTEM, and (d-f) element mapping of Mo and O of H-MoO_3_ nanosheets.

**Figure S11.** Element mapping of Ru, O, and Mo of Ru_1_@H-MoO_3-x_ nanosheets.

**Figure S12.** Wavelet transform of Mo foil, MoO_3_ reference, MoO_3_, H-MoO_3-x_, and Ru_1_@H-MoO_3-x_.

**Figure S13.** Wavelet transform of Ru foil, RuO_2_, and Ru_1_@H-MoO_3-x_.

**Figure S14.** Mott-Schottky plots of MoO_3_, H-MoO_3-x_, and Ru_1_@H-MoO_3-x_.

**Figure S15.** Schematic diagram of the band structure of MoO_3_, H-MoO_3-x_, and Ru_1_@H-MoO_3-x_.

**Figure S16.** The yield of H_2_ in photocatalytic CO_2_ reduction reaction on Ru_0.5_@H-MoO_3-x_, Ru_1_@H-MoO_3-x_ Ru_2_@H-MoO_3-x_, Ru_4_@H-MoO_3-x_ under full spectrum light irradiation.

**Figure S17.** Water contact angle over (a) MoO_3_, (b) H-MoO_3-x_, and (c) Ru_1_@H-MoO_3-x_.

**Figure S18.** Photocatalytic CO_2_ reduction product selectivity over H-MoO_3-x_, Ru_0.5_@H-MoO_3-x_, Ru_1_@H-MoO_3-x_ Ru_2_@H-MoO_3-x_, Ru_4_@H-MoO_3-x_ under (a) full spectrum light and (b) NIR light irradiation.

**Figure S19.** The turnover number (TON) of photocatalytic CO_2_ reduction over H-MoO_3-x_, Ru_0.5_@H-MoO_3-x_, Ru_1_@H-MoO_3-x_ Ru_2_@H-MoO_3-x_, Ru_4_@H-MoO_3-x_ under (a) full spectrum light and (b) NIR light irradiation.

**Figure S20.** Thermographic photographs of Ru_1_@H-MoO_3-x_ under light irradiation with different wavelengths of 420, 550, 600, and 700 nm.

**Figure S21.** (a) AQE of Ru_1_@H-MoO_3-x_ under light irradiation with different wavelengths of 420 and 700 nm. (b) the thermographic photographs of Ru_1_@H-MoO_3-x_ under 700 nm (0.7 W) light irradiation.

**Figure S22.** (a) XRD patterns and (b-d) XPS spectra of fresh and used Ru_1_@H-MoO_3-x_.

**Figure S23.** Thermographic photographs of Ru_1_@H-MoO_3-x_ under full spectrum irradiation. Temperature-time variation curves of MoO_3_, H-MoO_3-x_, and Ru_1_@H-MoO_3-x_.

**Figure S24.** PL spectra of H-MoO_3-x_ and Ru_1_@H-MoO_3-x_.

Figure S25. Calculated adsorption energy of CO_2_, CO, and CH_4_ on the surface of H-MoO_3-x_ and Ru@H-MoO_3-x_.

**Figure S26.** CO_2_ adsorption isotherms of H-MoO_3-x_ and Ru_1_@H-MoO_3-x_.

**3. Table Captions**

**Table S1.** The number of absolute spins of unpaired electrons in various samples.

**Table S2.** EXAFS fitting parameters at the Ru/Mo K-edge for various samples.

**Table S3.** Comparison of photocatalytic CO_2_ methanation performance with the reported various Mo-containing photocatalysts.

**Figure Captions**


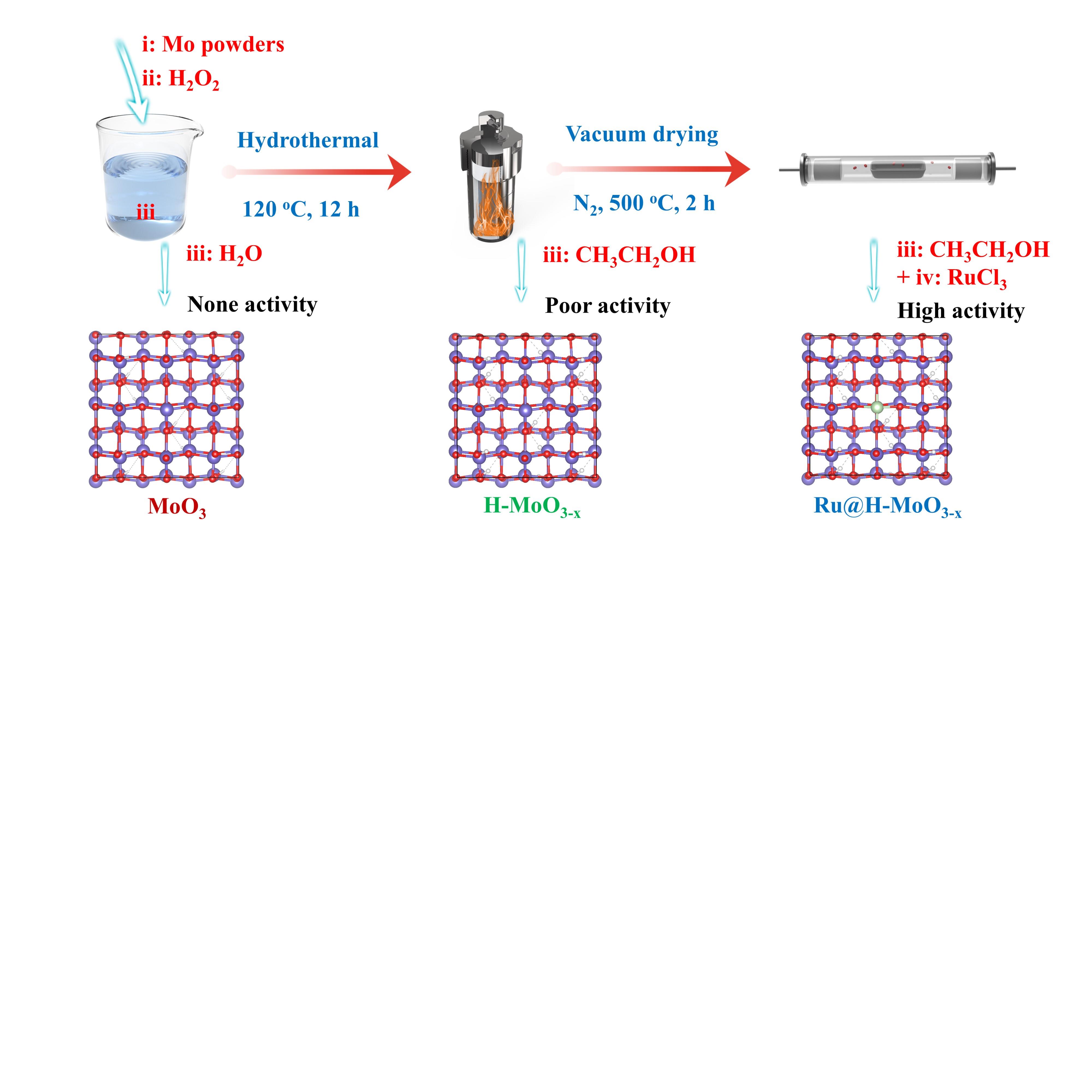


**Figure S1.** Synthetic diagram of MoO_3_, H-MoO_3-x_, and Ru@H-MoO_3-x_ nanosheets.


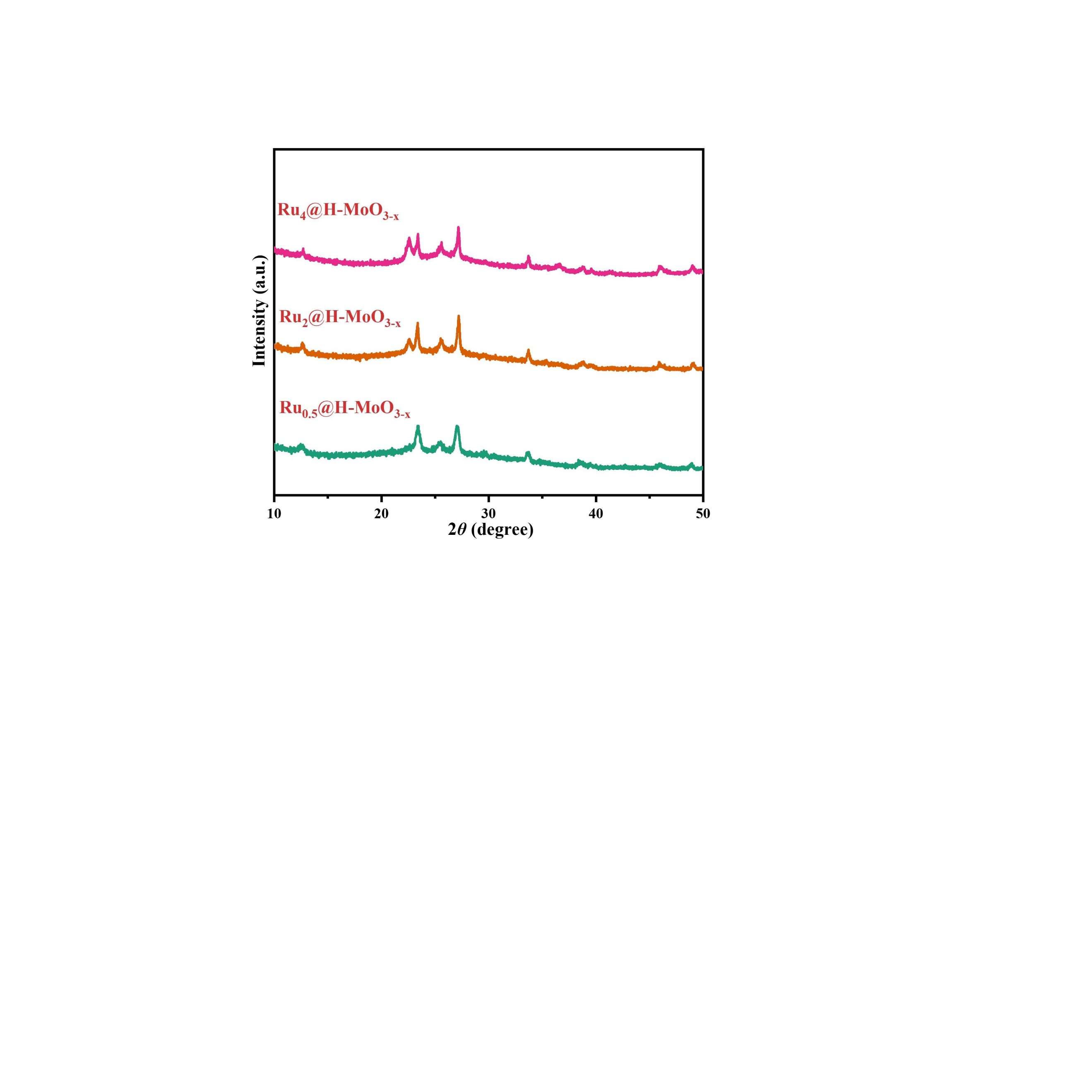


**Figure S2.** XRD patterns of Ru@H-MoO_3-x_ nanosheets.


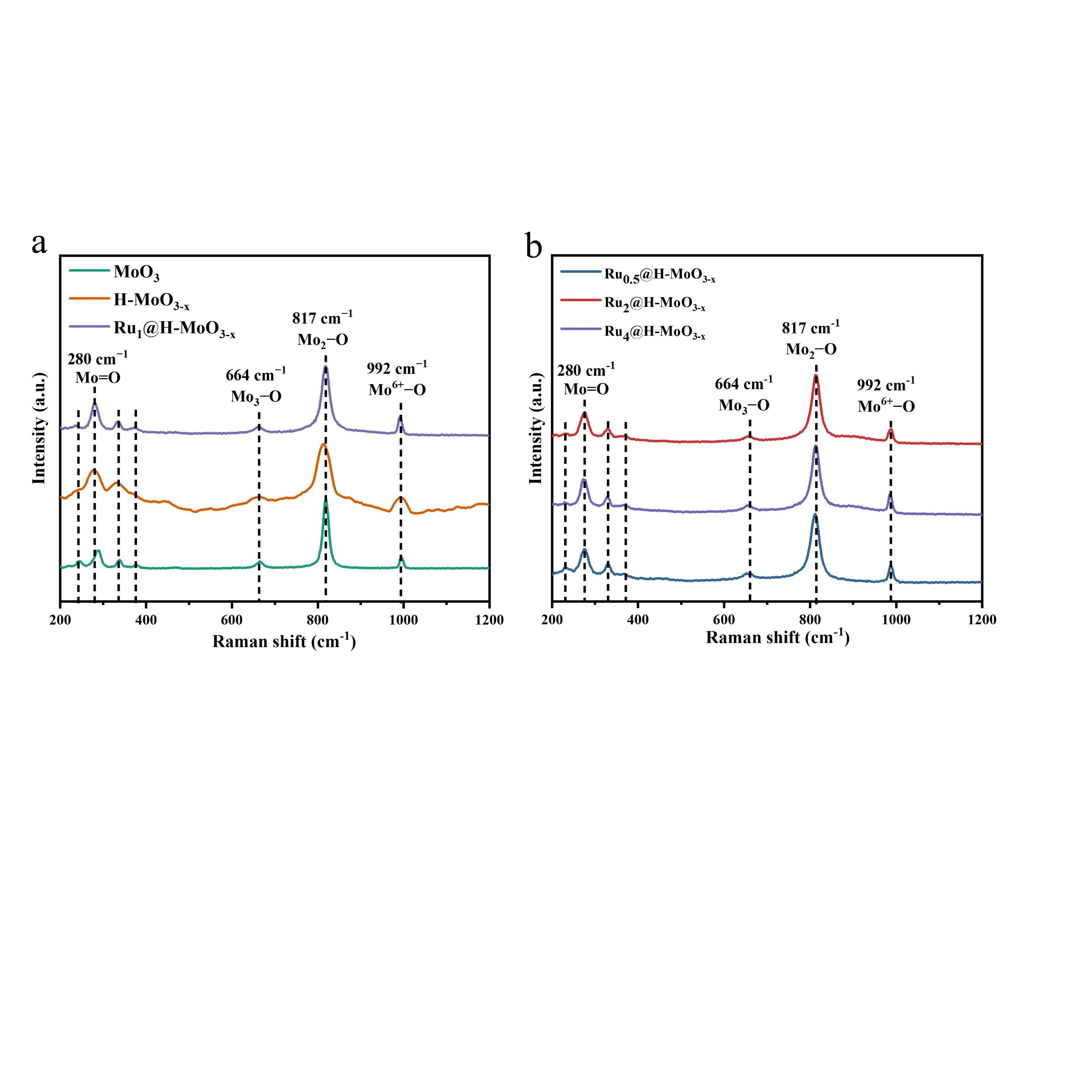


**Figure S3.** Raman spectra of MoO_3_, H-MoO_3-x_, and Ru@H-MoO_3-x_.


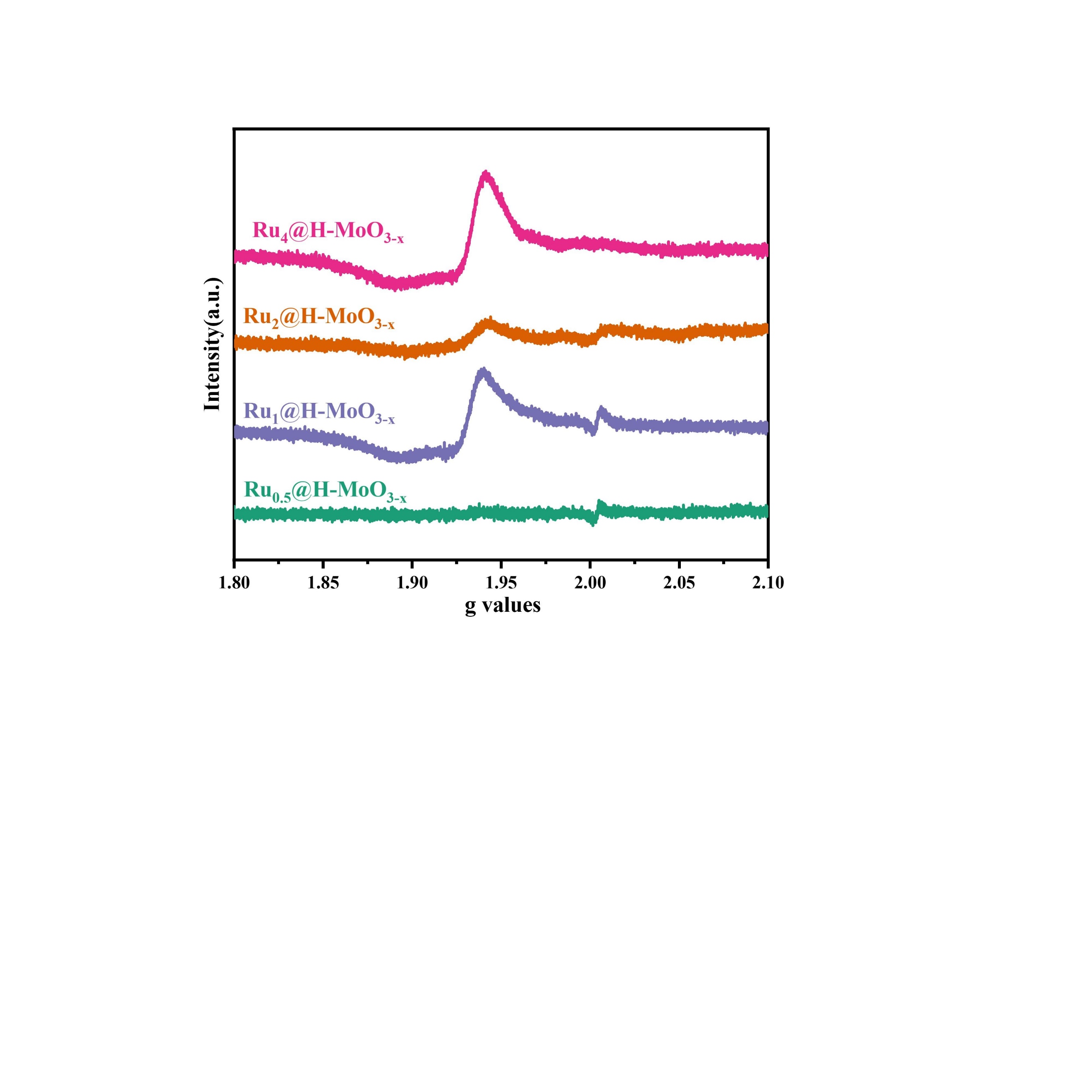


**Figure S4.** ESR spectra of Ru@H-MoO_3-x_ nanosheets.


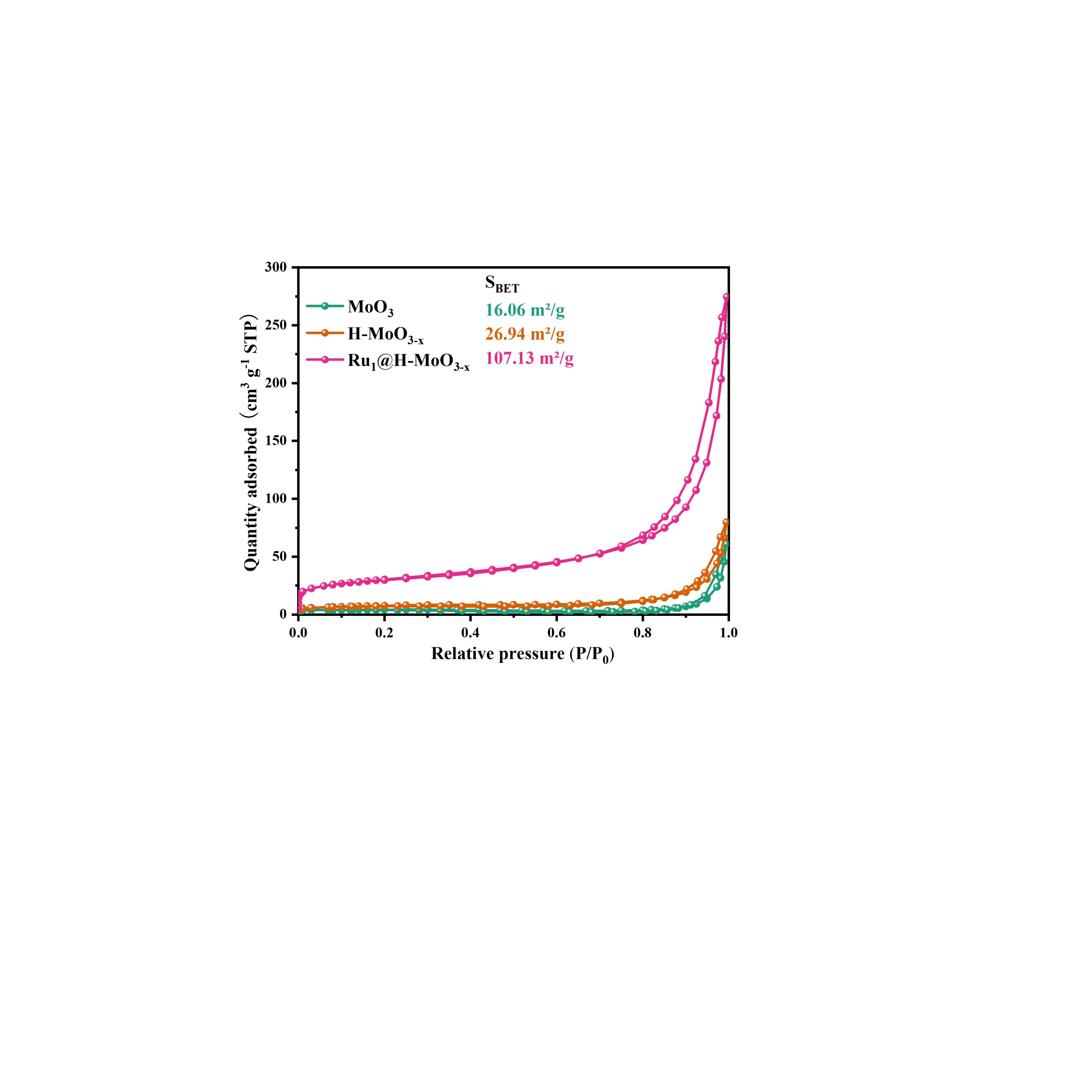


**Figure S5.** N_2_ adsorption/desorption isotherms of MoO_3_, H-MoO_3-x_, and Ru@H-MoO_3-x_ nanosheets.


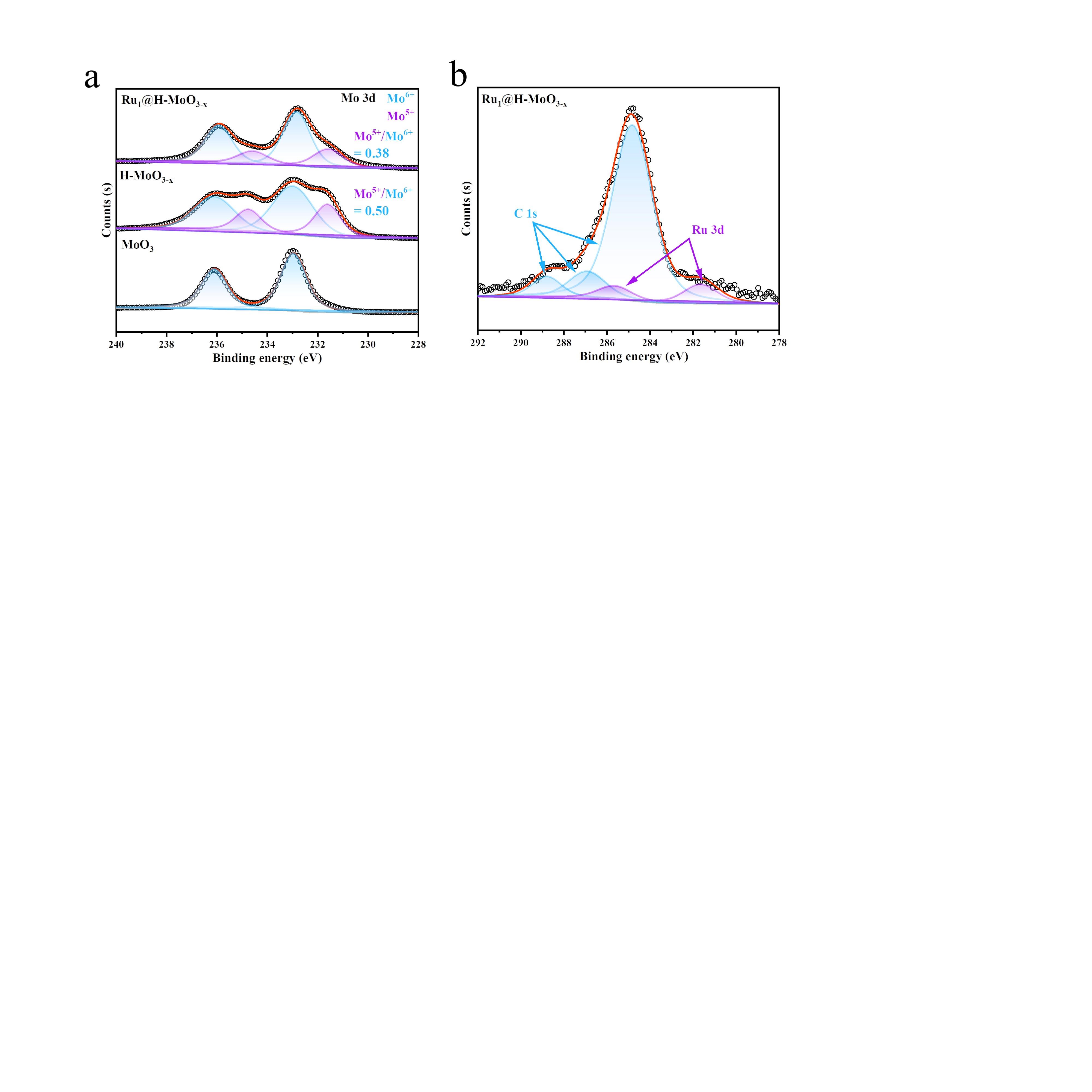


**Figure S6.** XPS spectra of MoO_3_, H-MoO_3-x_, and Ru_1_@H-MoO_3-x_: (a) Mo 3d, (b) Ru 3d.


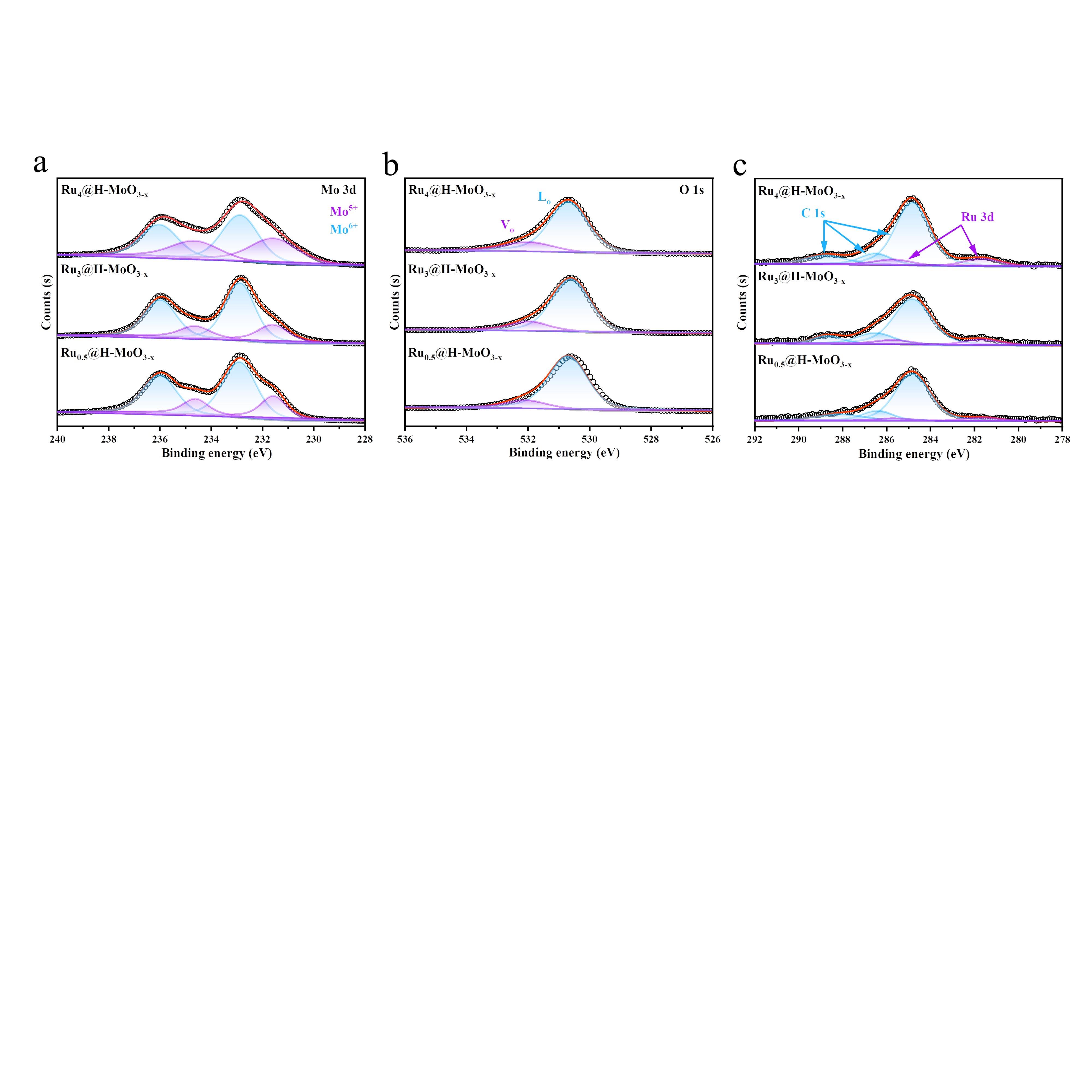


**Figure S7.** XPS spectra for a series of Ru@H-MoO_3-x_: (a) Mo 3d, (b) O 1s, (c) Ru 3d and C 1s.


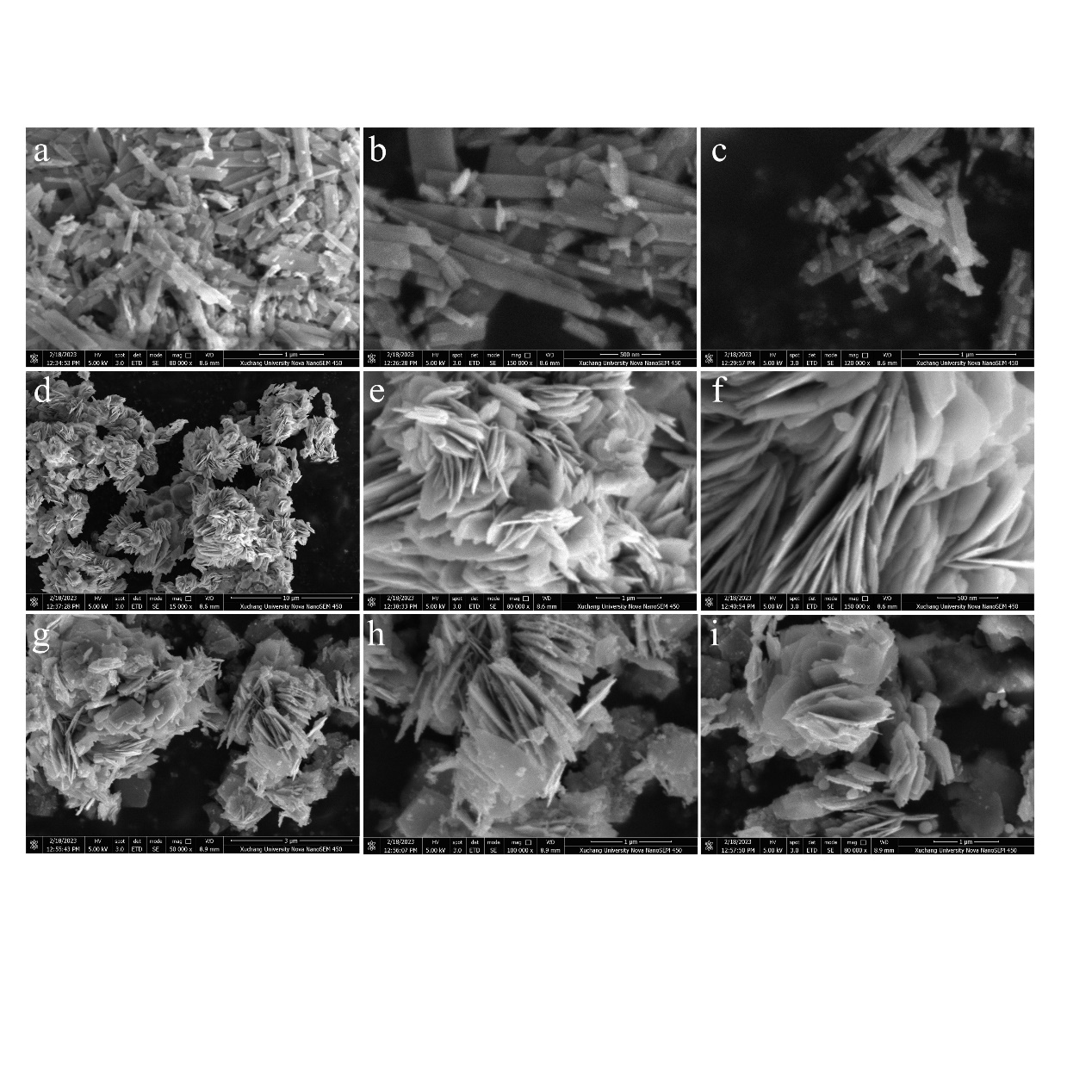


**Figure S8.** SEM images of (a-c) MoO_3_, (d-f) H-MoO_3,_ and (g-i) Ru_1_@H-MoO_3_ nanosheets.


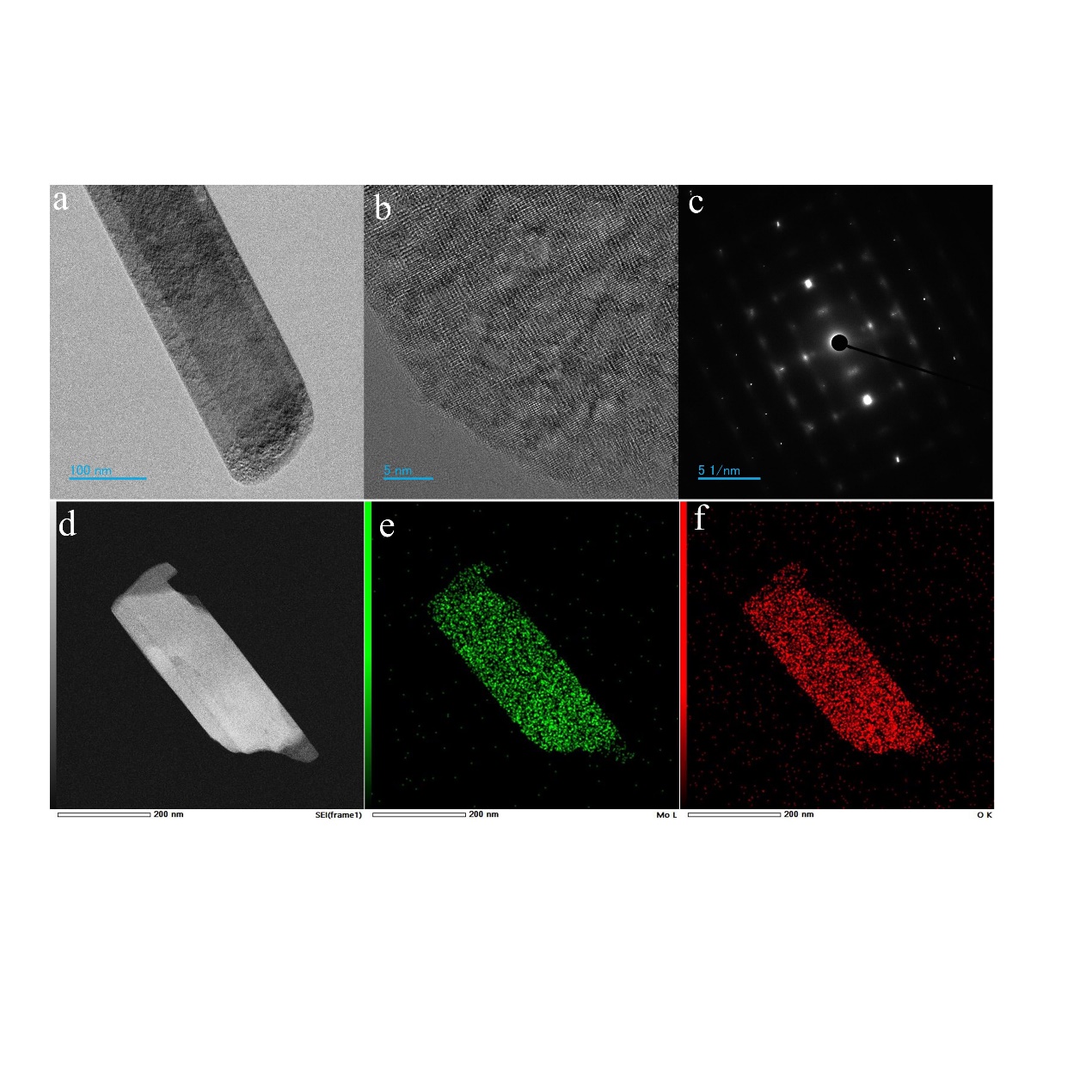


**Figure S9.** (a) TEM, (b) HRTEM, (c) SADE, and (d-f) element mapping of Mo and O of MoO_3_ nanosheets.


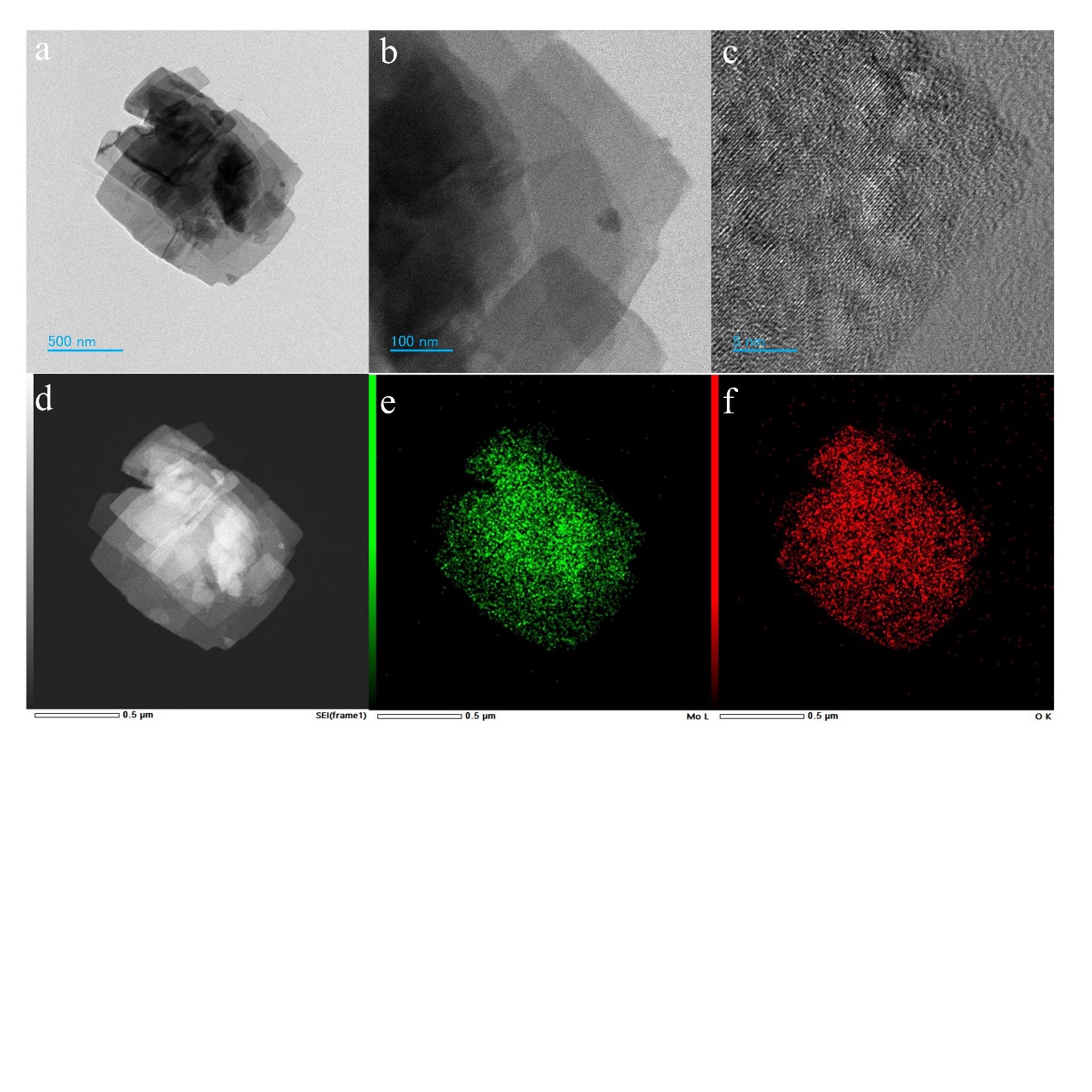


**Figure S10.** (a-b) TEM, (c) HRTEM, and (d-f) element mapping of Mo and O of H-MoO_3_ nanosheets.


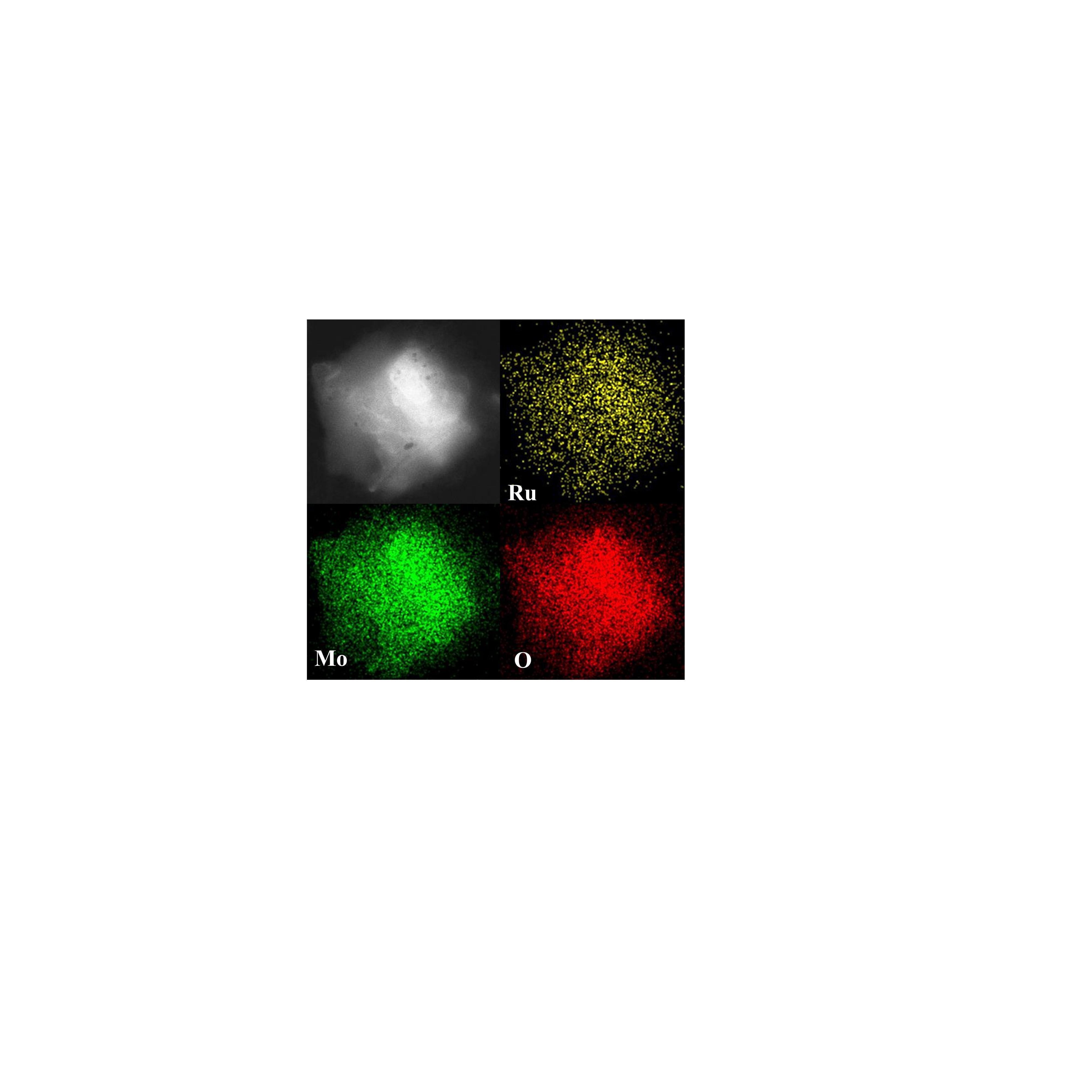


**Figure S11.** Element mapping of Ru, O, and Mo of Ru_1_@H-MoO_3-x_ nanosheets.


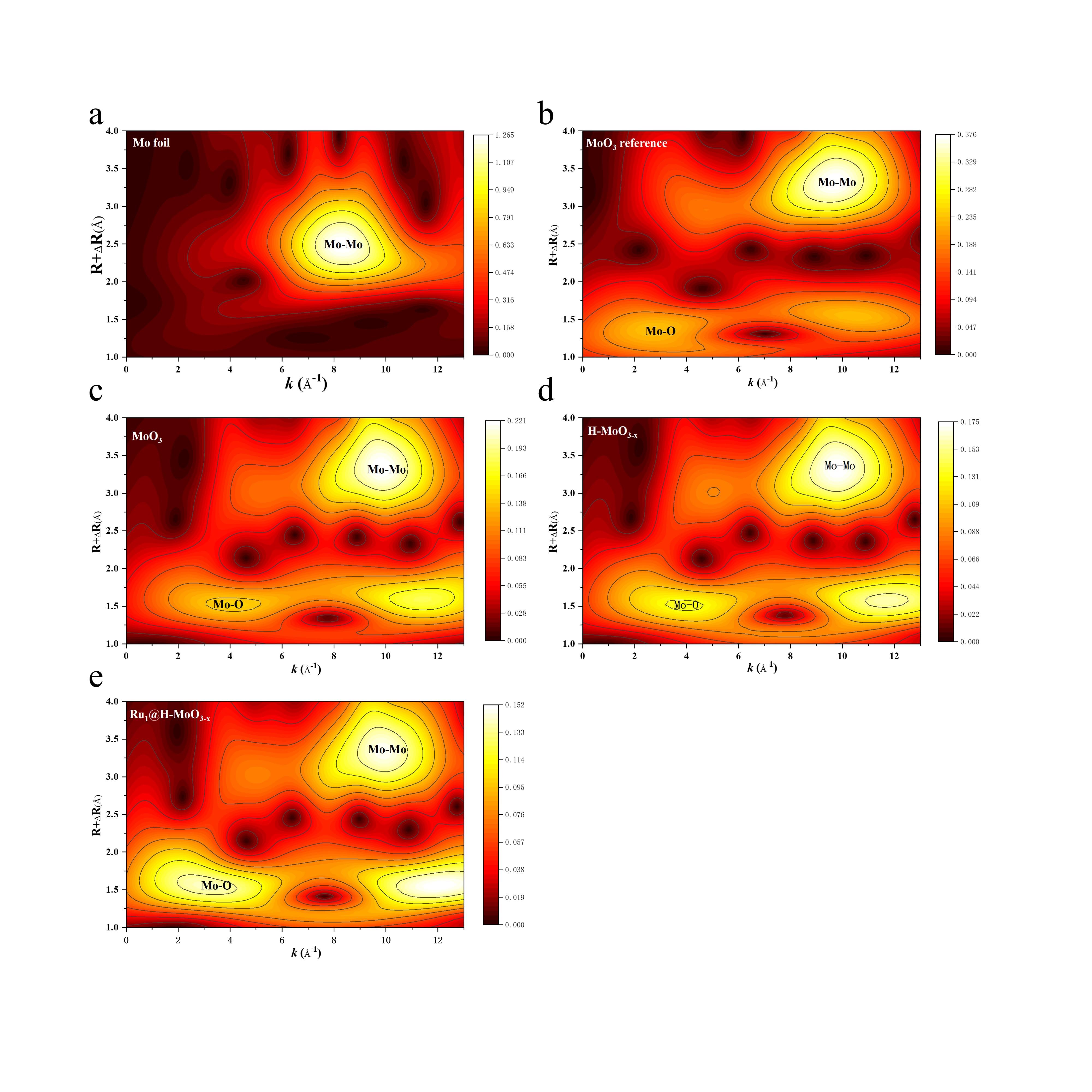


**Figure S12.** Wavelet transform of Mo foil, MoO_3_ reference, MoO_3_, H-MoO_3-x_, and Ru_1_@H-MoO_3-x_.


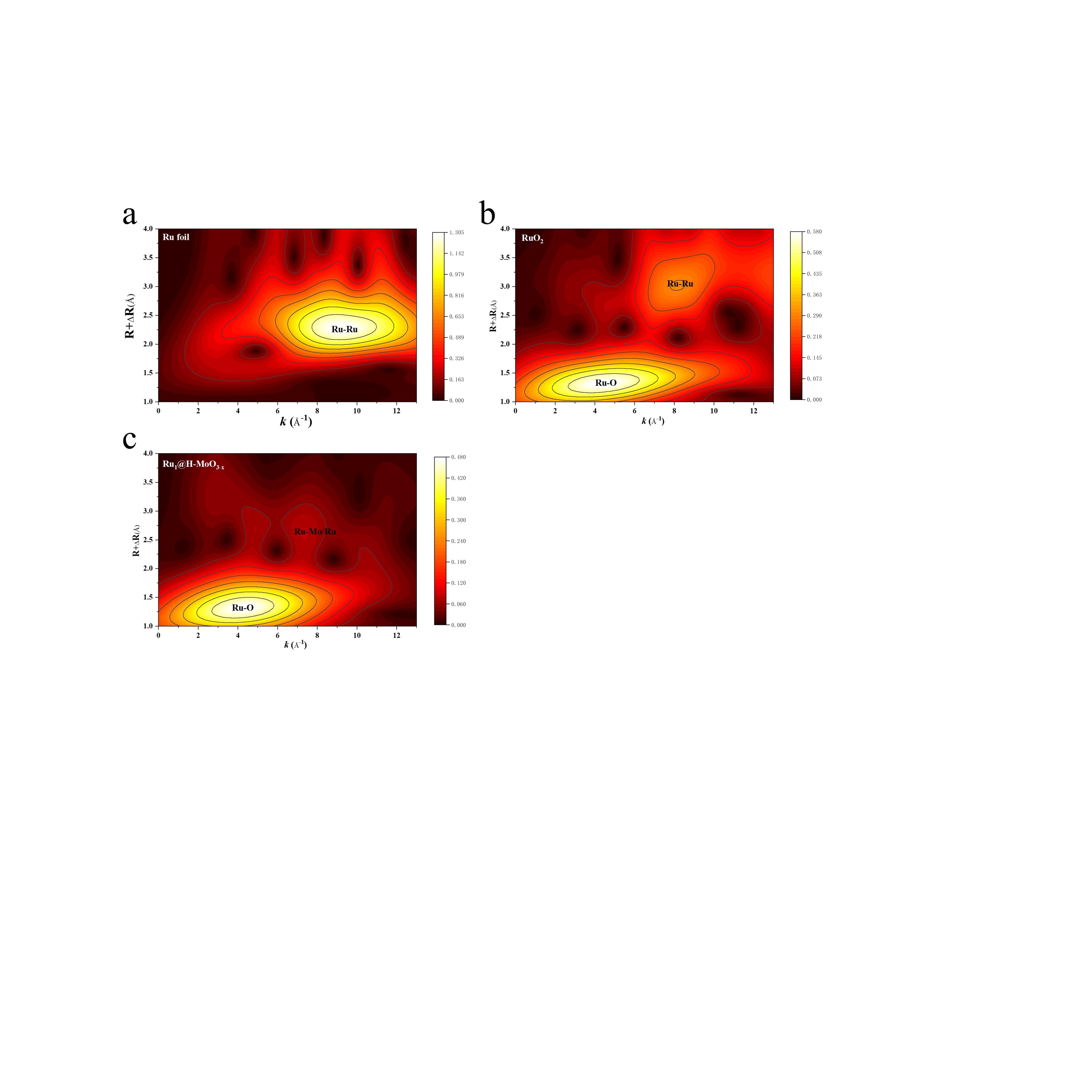


**Figure S13.** Wavelet transform of Ru foil, RuO_2_, and Ru_1_@H-MoO_3-x_.


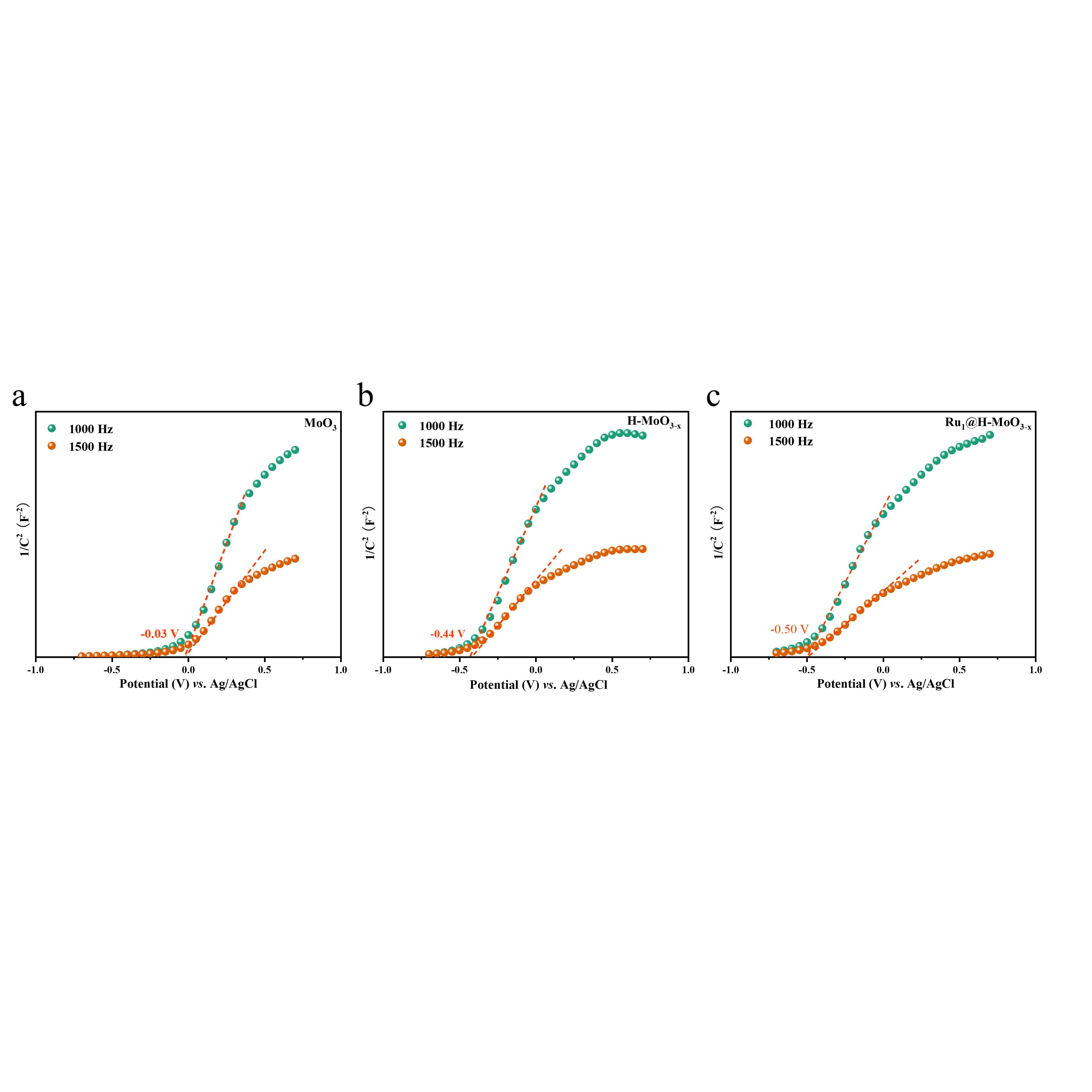


**Figure S14.** Mott-Schottky plots of MoO_3_, H-MoO_3-x_, and Ru_1_@H-MoO_3-x_.


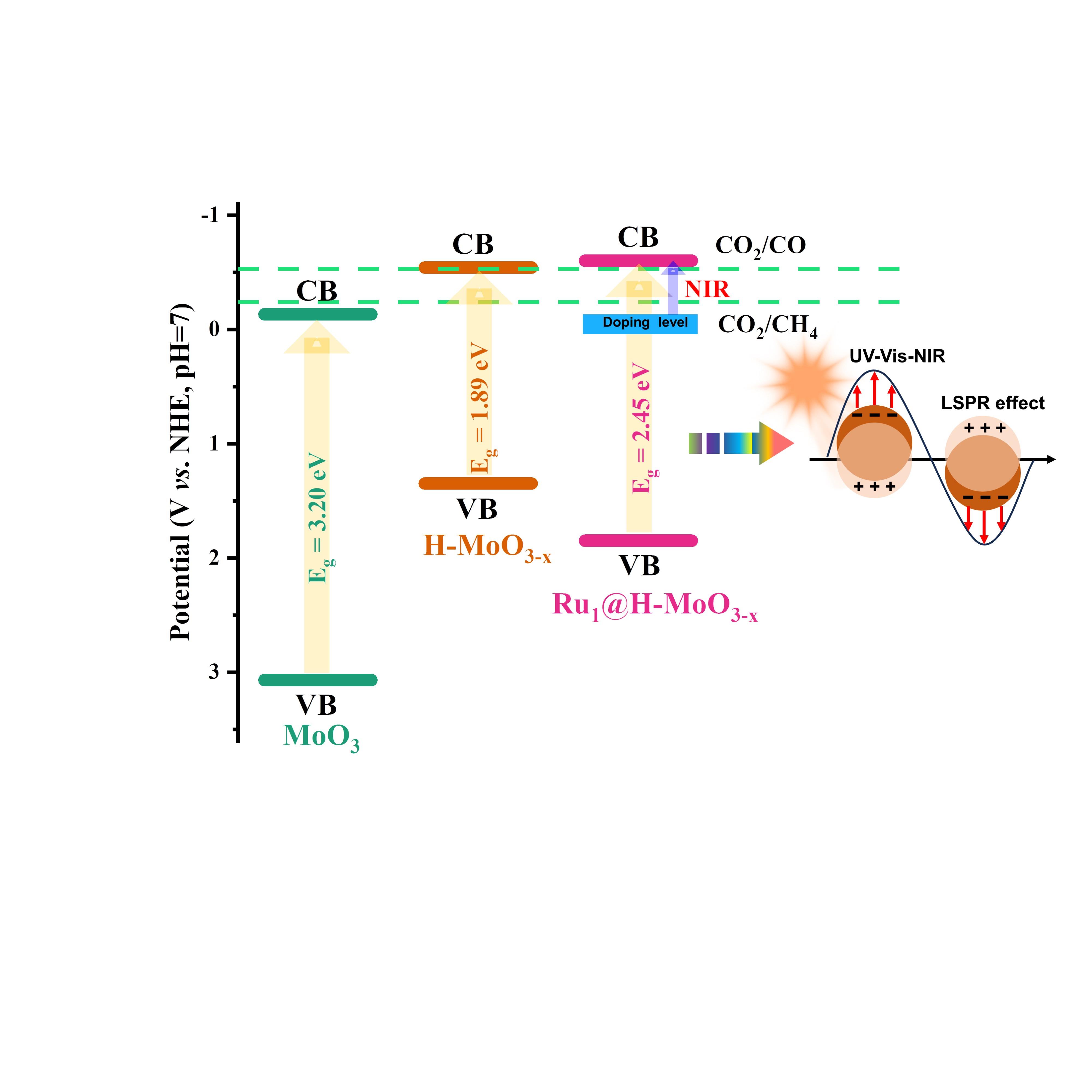


**Figure S15.** Schematic diagram of the band structure of the samples.


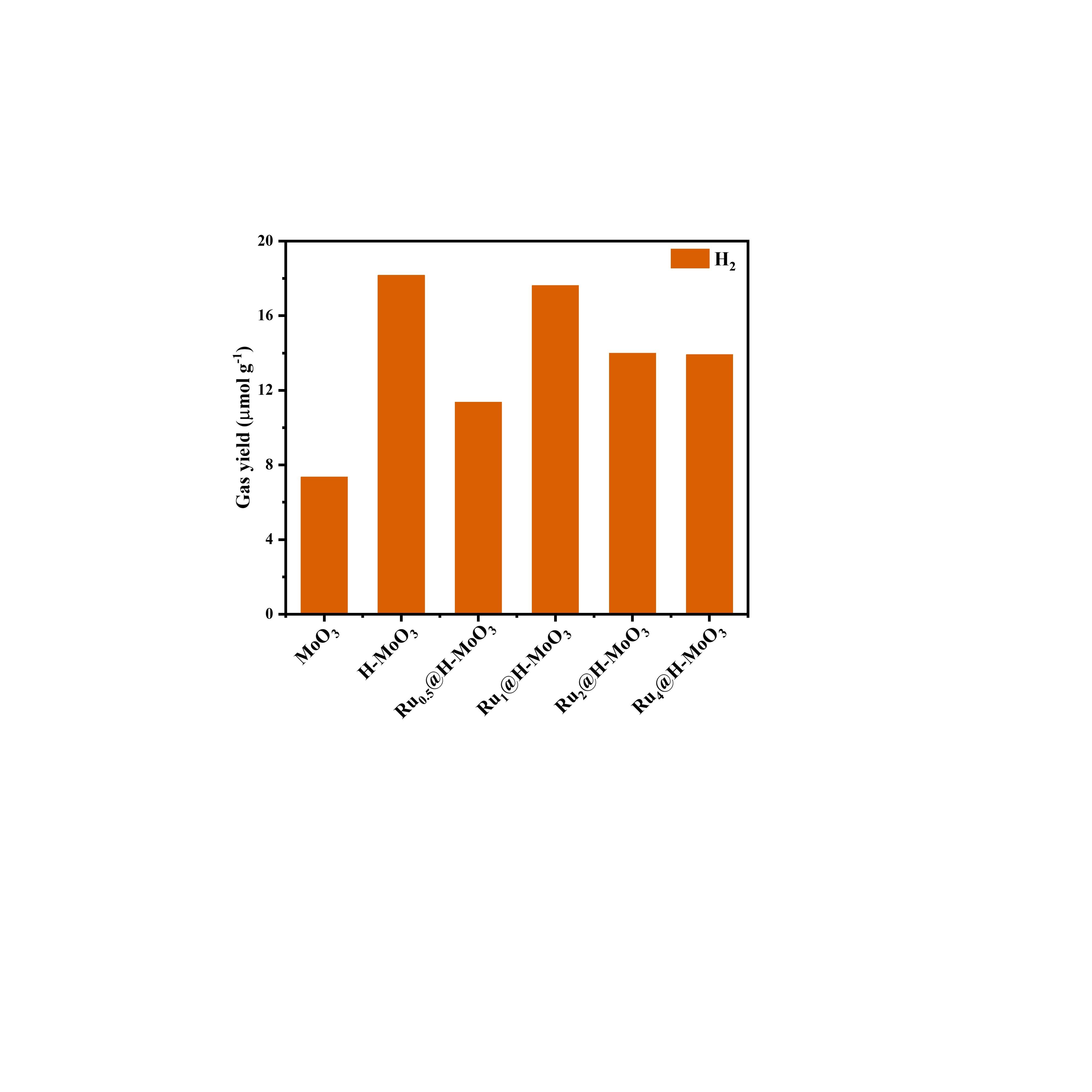


**Figure S16.** The yield of H_2_ in photocatalytic CO_2_ reduction reaction on Ru_0.5_@H-MoO_3-x_, Ru_1_@H-MoO_3-x_ Ru_2_@H-MoO_3-x_, Ru_4_@H-MoO_3-x_ under full spectrum light irradiation.


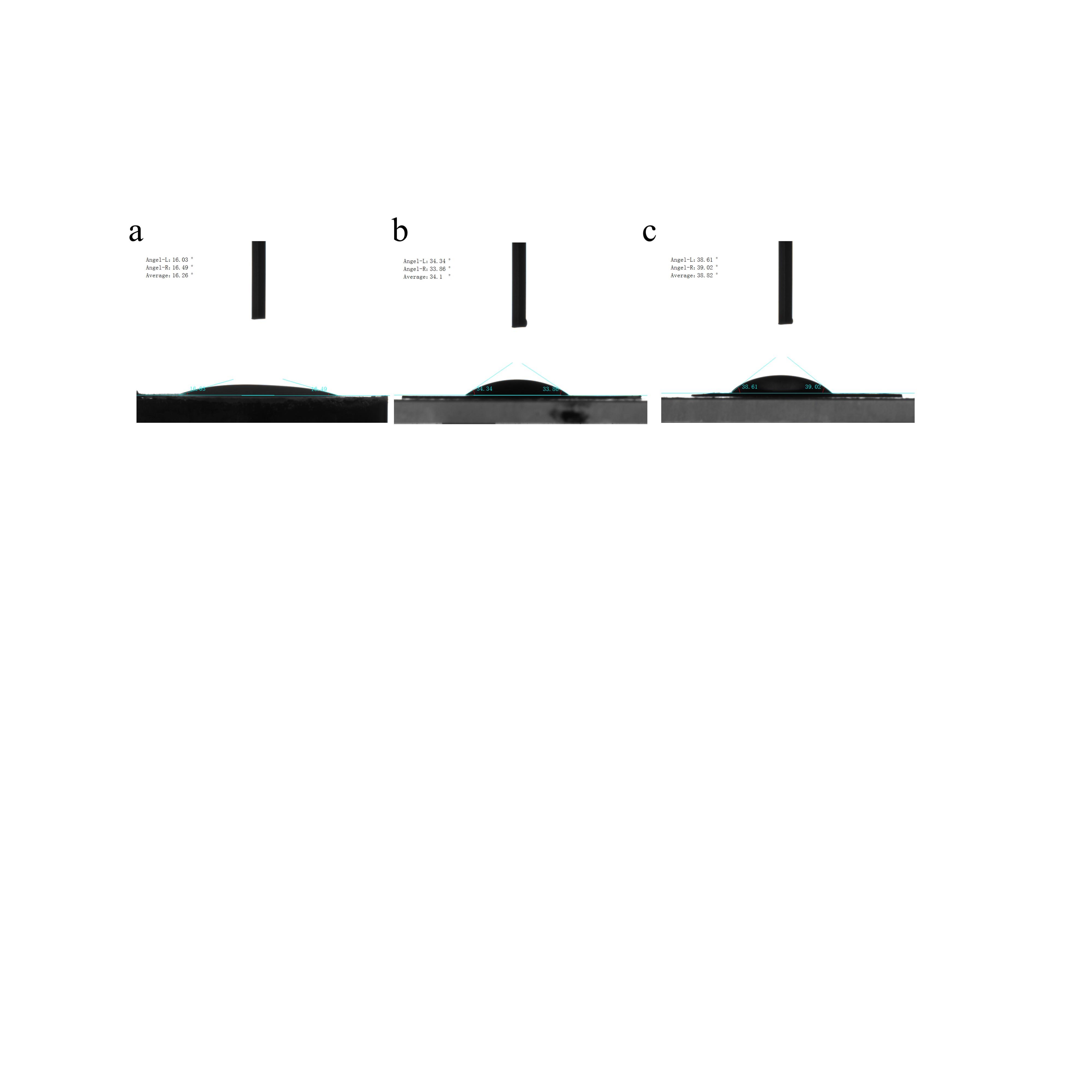


**Figure S17.** Water contact angle over (a) MoO_3_, (b) H-MoO_3-x_, and (c) Ru_1_@H-MoO_3-x_.


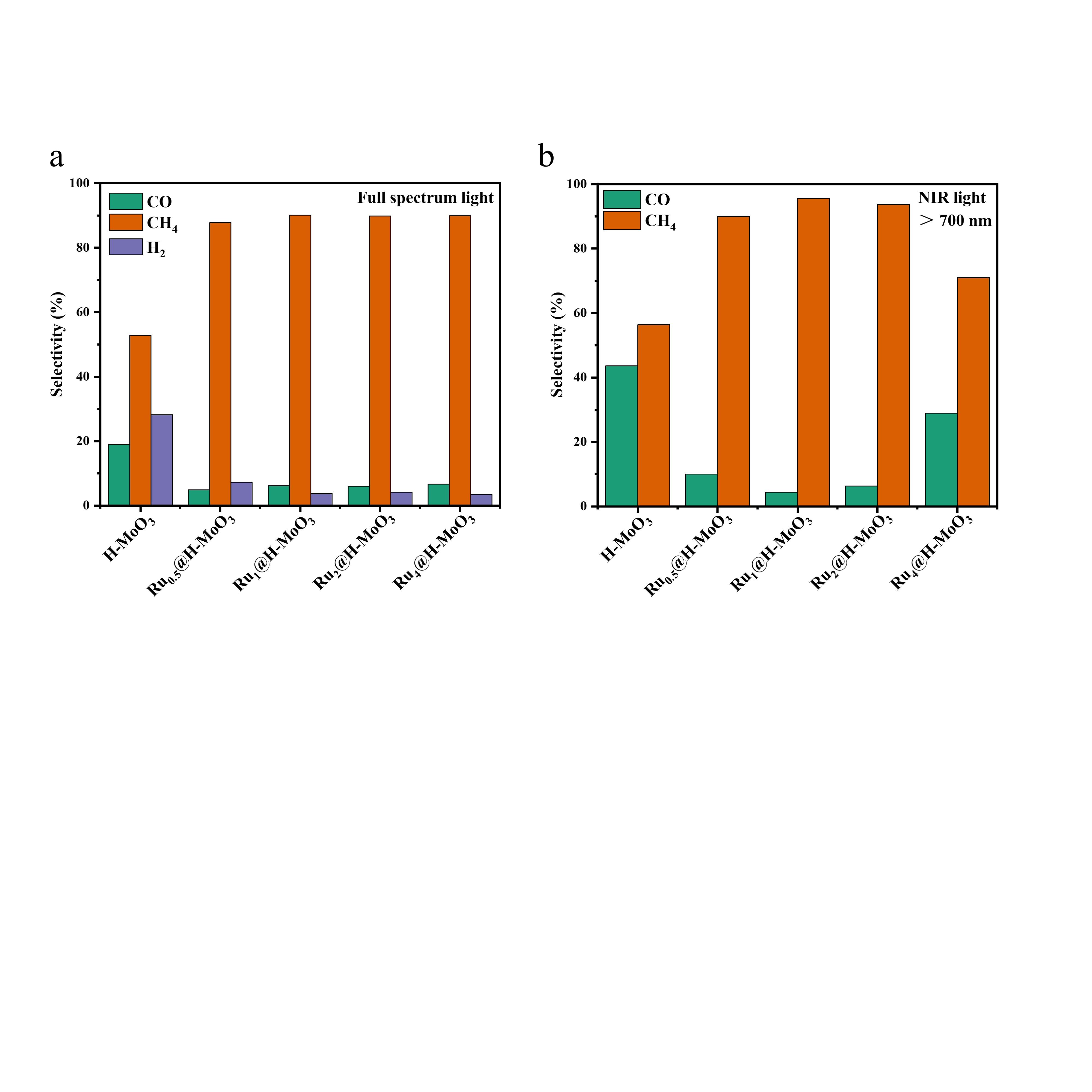


**Figure S18.** Photocatalytic CO_2_ reduction product selectivity over H-MoO_3-x_, Ru_0.5_@H-MoO_3-x_, Ru_1_@H-MoO_3-x_ Ru_2_@H-MoO_3-x_, Ru_4_@H-MoO_3-x_ under (a) full spectrum light and (b) NIR light irradiation.


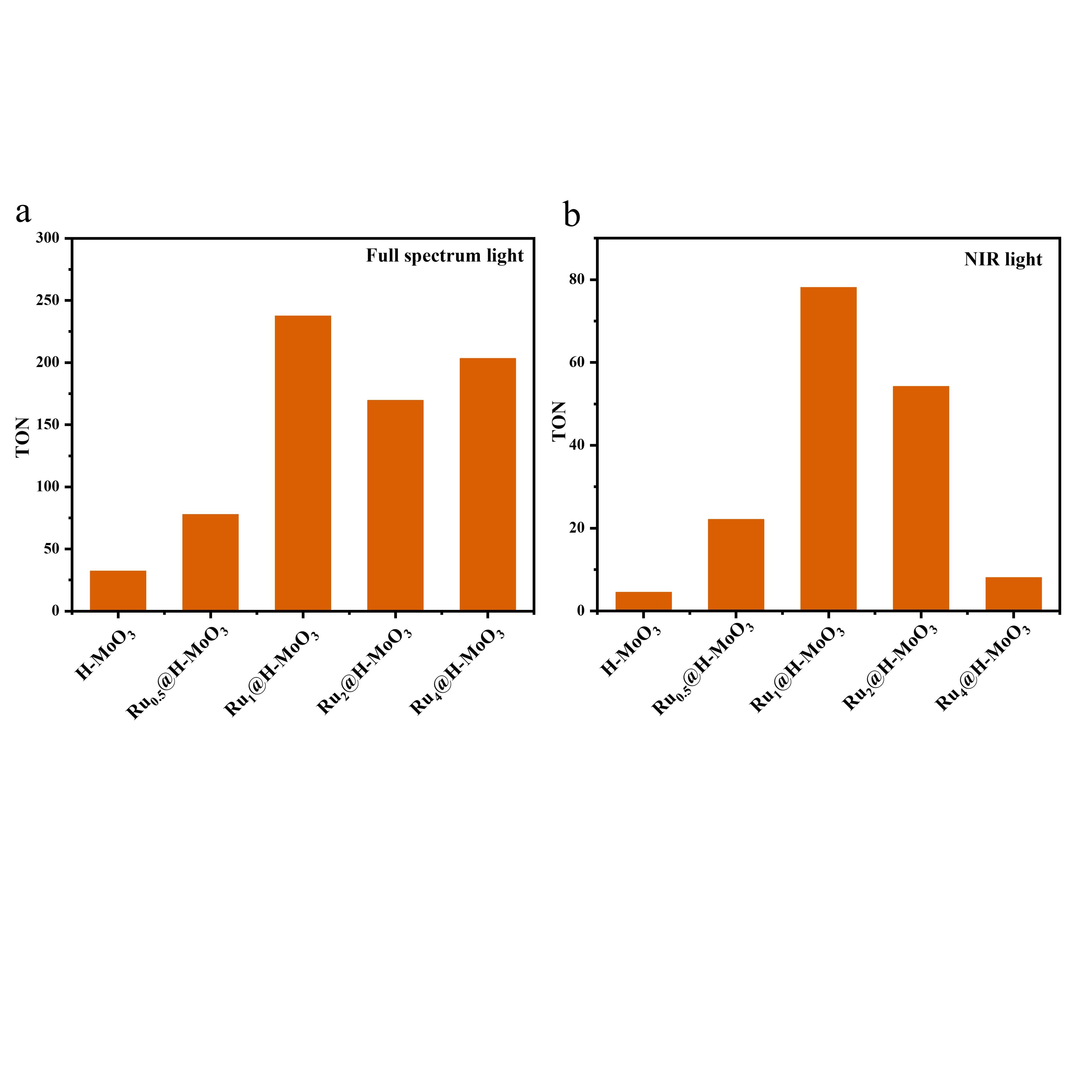


**Figure S19.** The turnover number (TON) of photocatalytic CO_2_ reduction over H-MoO_3-x_, Ru_0.5_@H-MoO_3-x_, Ru_1_@H-MoO_3-x_ Ru_2_@H-MoO_3-x_, Ru_4_@H-MoO_3-x_ under (a) full spectrum light and (b) NIR light irradiation.


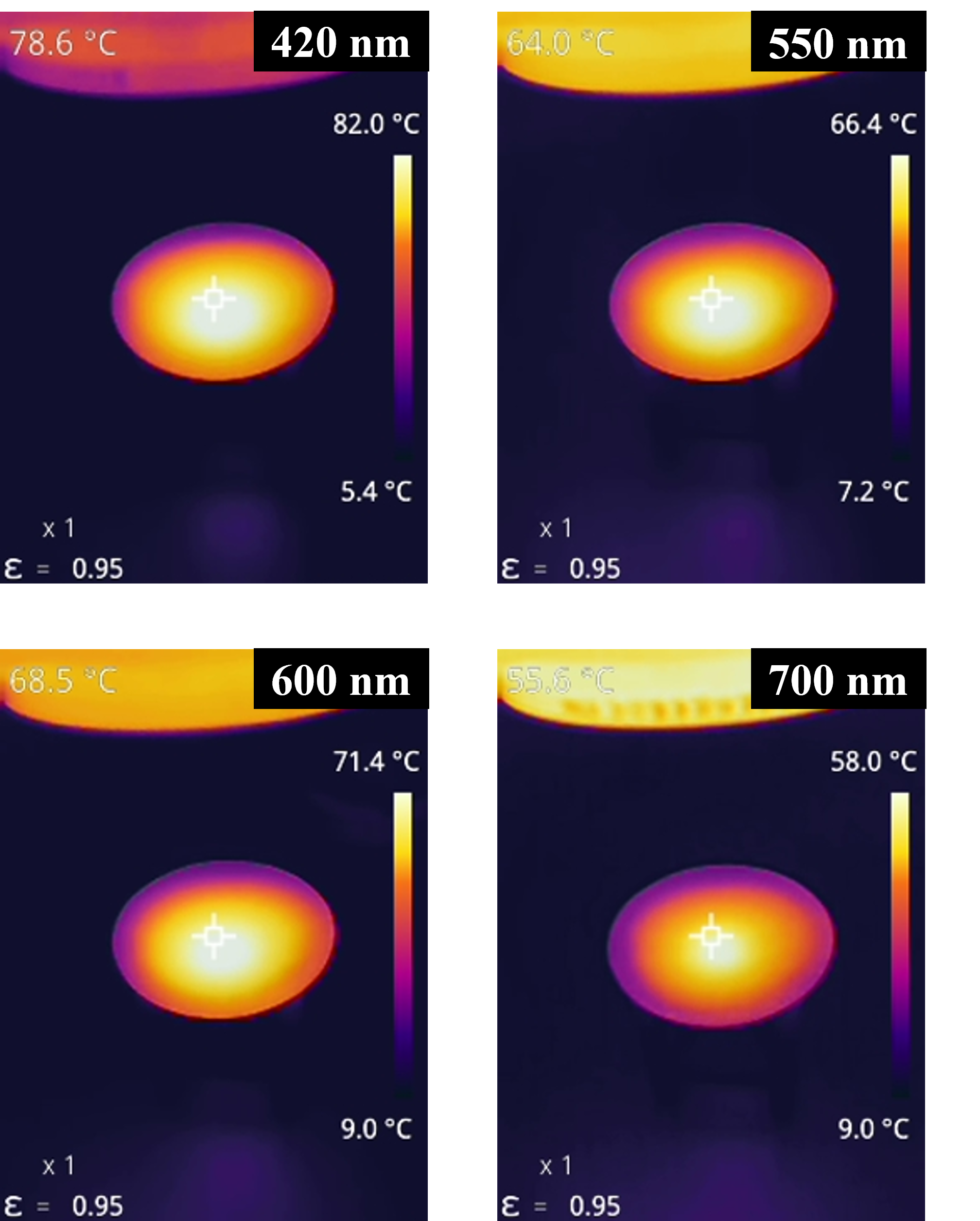


**Figure S20.** Thermographic photographs of Ru_1_@H-MoO_3-x_ under light irradiation with different wavelengths of 420, 550, 600, and 700 nm.


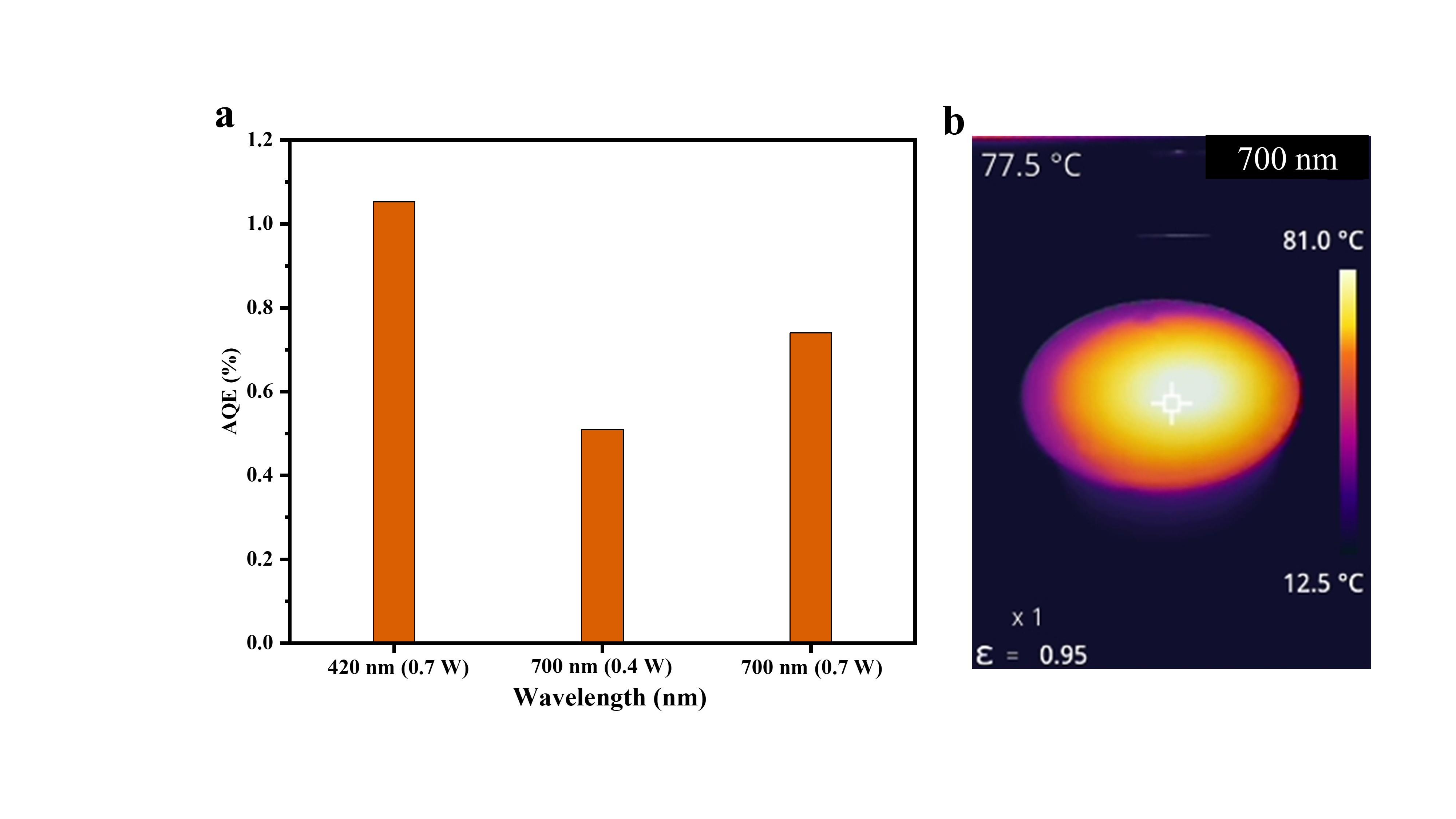


**Figure S21.** (a) AQE of Ru_1_@H-MoO_3-x_ under light irradiation with different wavelengths of 420 and 700 nm. (b) the thermographic photographs of Ru_1_@H-MoO_3-x_ under 700 nm (0.7 W) light irradiation.


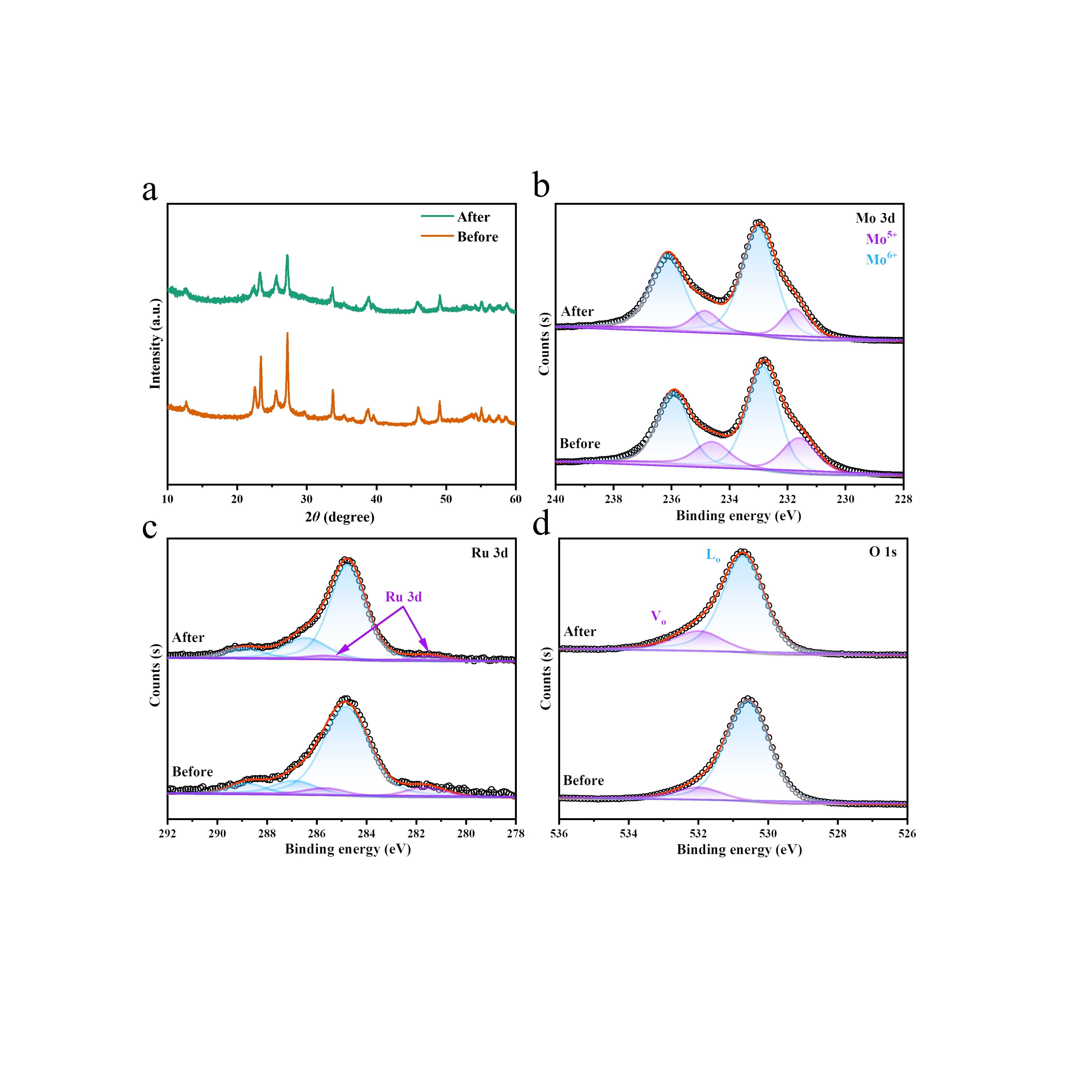


**Figure S22.** (a) XRD patterns and (b-d) XPS spectra of fresh and used Ru_1_@H-MoO_3-x_.


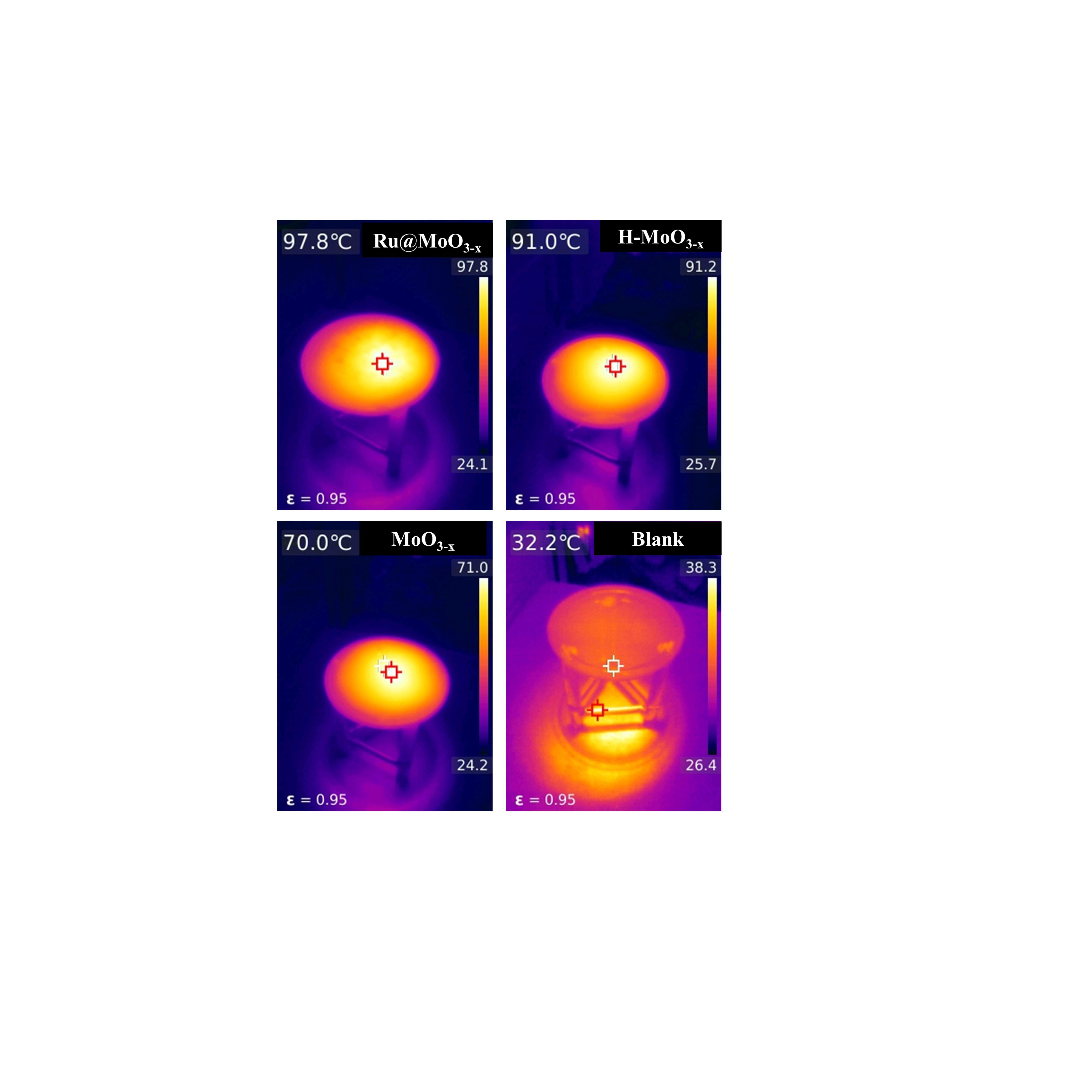


Figure S23. Thermographic photographs of Ru_1_@H-MoO_3-x_ under full spectrum irradiation. Temperature-time variation curves of MoO_3_, H-MoO_3-x_, and Ru_1_@H-MoO_3-x_.


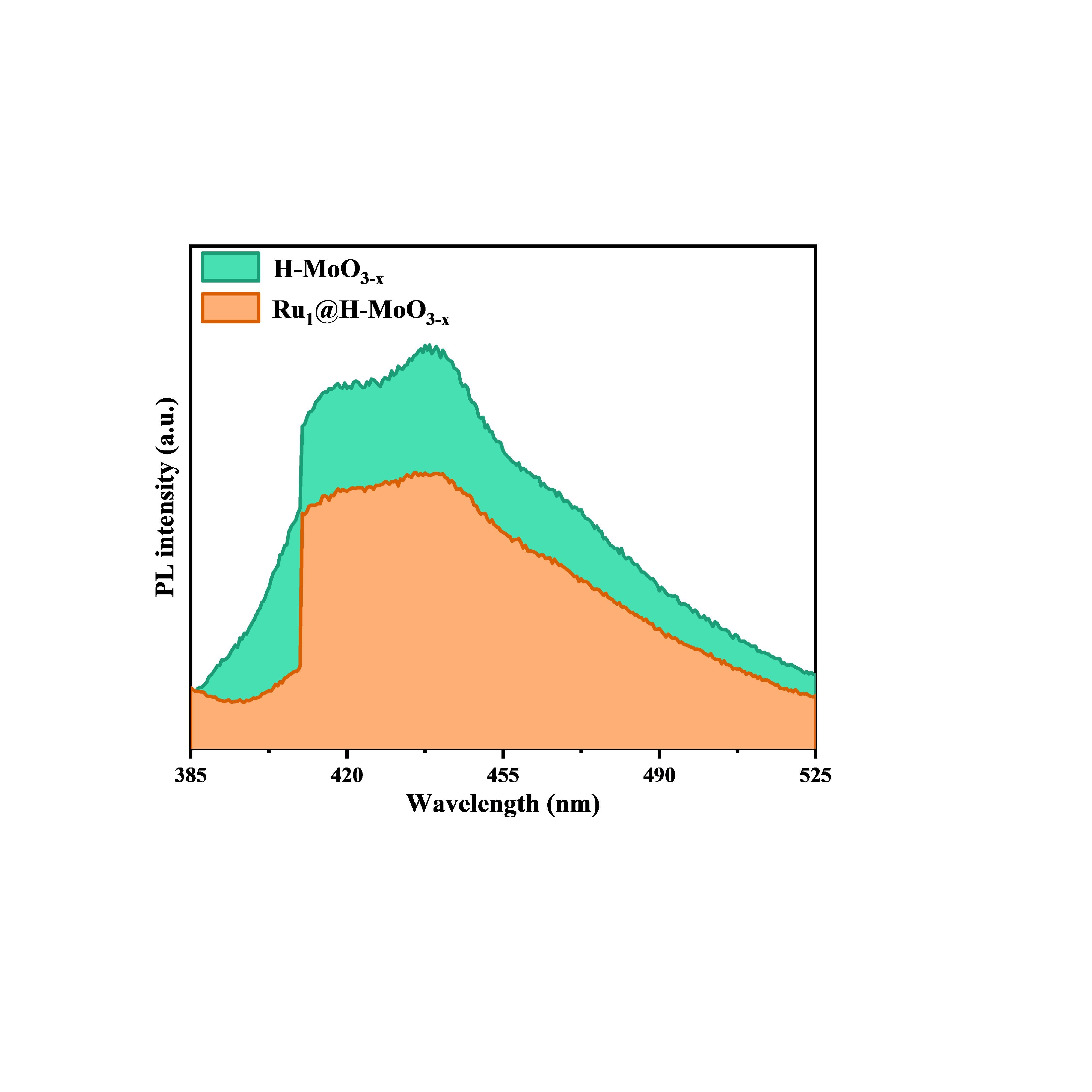


**Figure S24.** PL spectra of H-MoO_3-x_ and Ru_1_@H-MoO_3-x_.


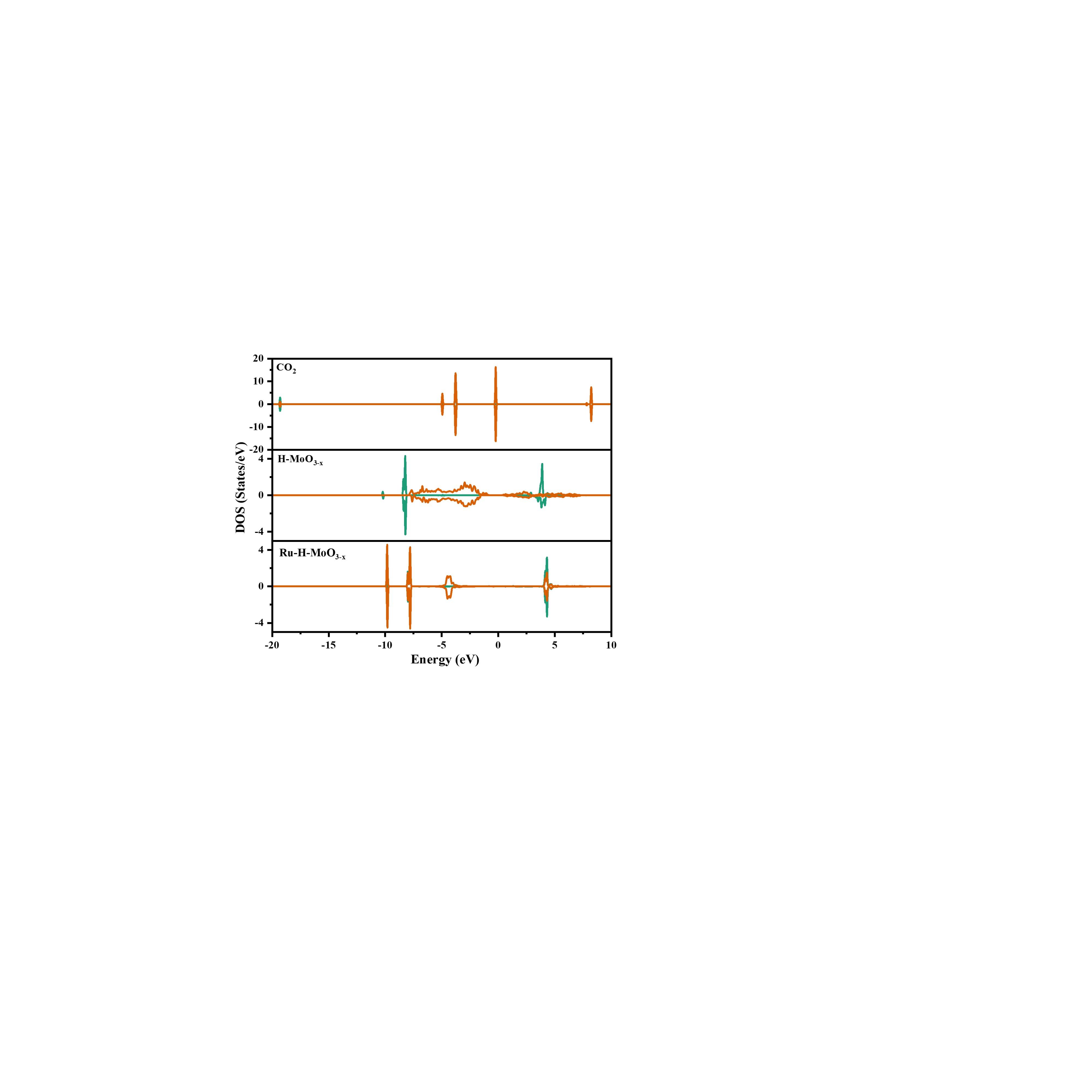


Figure S25. Calculated adsorption energy of CO_2_, CO, and CH_4_ on the surface of H-MoO_3-x_ and Ru@H-MoO_3-x_.


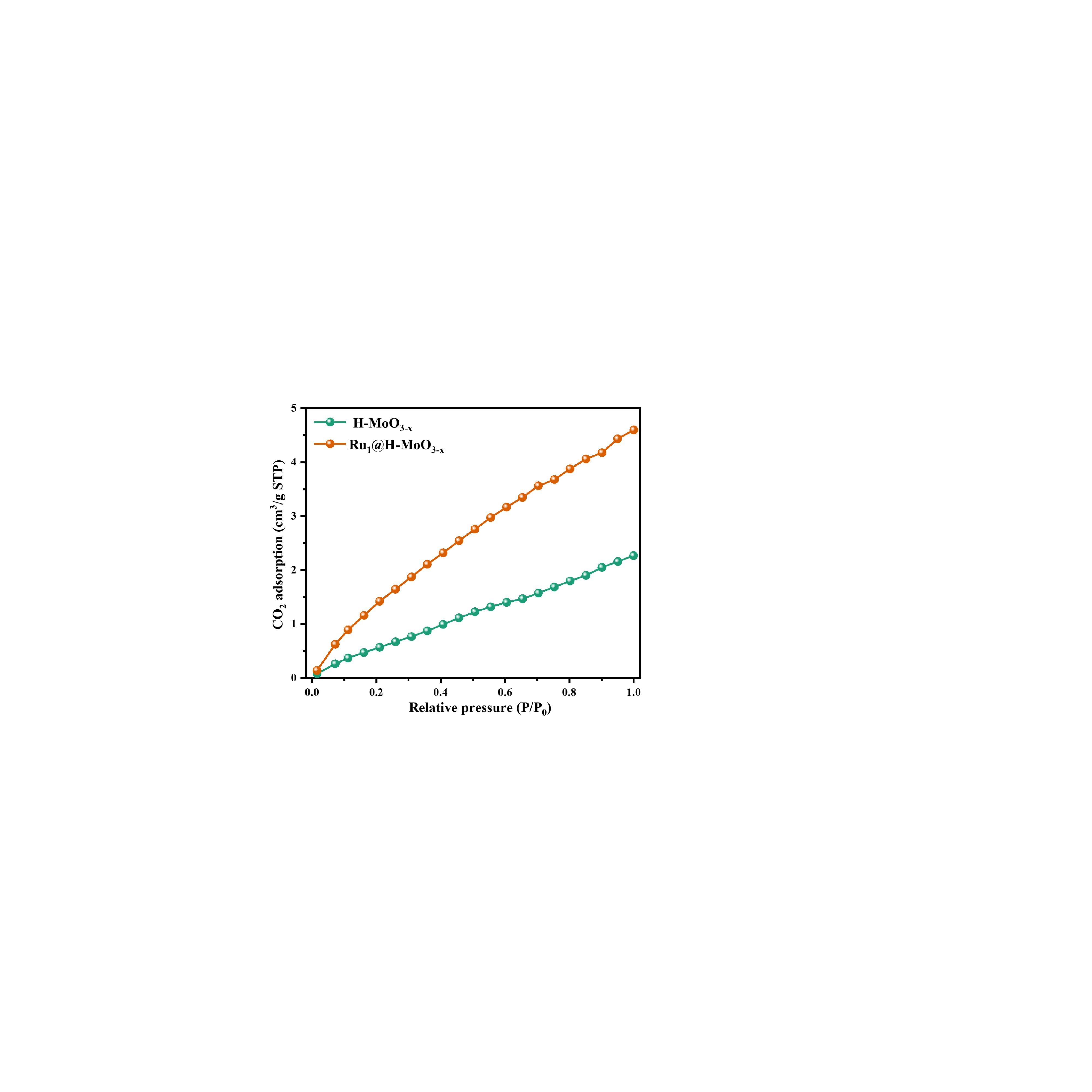


**Figure S26.** CO_2_ adsorption isotherms of H-MoO_3-x_ and Ru_1_@H-MoO_3-x_.

**Table S1.** The number of absolute spins of unpaired electrons in various samples.

| Samples | Spins/mm^3^ |
| --- | --- |
| MoO_3_ | 0 |
| H-MoO_3-x_ | 1.107e+14 |
| Ru_1_@H-MoO_3-x_ | 4.095e+13 |

**Table S2.** EXAFS fitting parameters at the Ru/Mo K-edge for various samples.

| Sample | Shell | *CN^a^* | *R*(Å)*^b^* | *σ*^2^(Å^2^)*^c^* | Δ*E*_0_(eV)*^d^* | *R* factor |
| --- | --- | --- | --- | --- | --- | --- |
| Ru K-edge (*S*_0_^2^ = 0.881) | | | | | | |
| Ru foil | Ru-Ru | 12* | 2.675±0.002 | 0.0041±0.0003 | -4.7±0.7 | 0.0042 |
| RuO_2_ | Ru-O | 6.0±0.3 | 1.976±0.003 | 0.0032±0.0004 | -0.6±1.0 | 0.0040 |
|  | Ru-Ru | 3.9±0.5 | 3.135±0.004 | 0.0100±0.0012 | 2.5±1.3 |  |
|  | Ru-Ru | 7.9±0.7 | 3.577±0.009 |  | -4.8±1.6 |  |
| Ru_1_@H-MoO_3-x_ | Ru-O | 5.1±0.3 | 1.991±0.011 | 0.0081±0.0015 | 3.5±2.0 | 0.0077 |
|  | Ru-Mo/Ru | 2.6±0.5 | 3.107±0.010 | 0.0100±0.0037 | 2.6±7.7 |  |
| Mo K-edge (*S*_0_^2^ = 0.871) | | | | | | |
| Mo foil | Mo-Mo | 8* | 2.721±0.003 | 0.0040±0.0003 | 5.2±0.9 | 0.0039 |
|  | Mo-Mo | 6* | 3.139±0.005 | 0.0040±0.0004 |  |  |
| MoO_3_ reference | Mo-O | 6.0±0.2 | 1.988±0.010 | 0.0062±0.0029 | 8.4±0.6 | 0.0054 |
|  | Mo-Mo | 5.1±0.6 | 3.698±0.008 | 0.0073±0.0020 | -7.3±3.5 |  |
|  | Mo-Mo | 3.6±0.5 | 3.921±0.009 |  | 4.6±0.2 |  |
| H-MoO_3-x_ | Mo-O | 5.9±0.3 | 2.096±0.002 | 0.0081±0.0012 | 2.7±4.6 | 0.0051 |
|  | Mo-Mo | 6.9±0.5 | 3.717±0.011 | 0.0080±0.0045 | -1.7±5.0 |  |
| 211-Mo-2 | Mo-O | 5.3±0.3 | 2.106±0.006 | 0.0082±0.0023 | 5.9±9.8 | 0.0018 |
|  | Mo-Mo | 5.5±0.8 | 3.708±0.016 | 0.0199±0.0151 | -2.5±6.8 |  |
| Ru_1_@H-MoO_3-x_ | Mo-O | 4.7±0.3 | 2.103±0.011 | 0.0079±0.0036 | 0.8±5.0 | 0.0026 |
|  | Mo-Mo | 4.3±0.6 | 3.707±0.017 | 0.0184±0.0092 | -3.3±6.5 |  |

*^a^CN*, coordination number; *^b^R*, the distance to the neighboring atom; *^c^σ*^2^, the Mean Square Relative Displacement (MSRD); *^d^ΔE*_0_, inner potential correction; *R* factor indicates the goodness of the fit. *S*0^2^ was fixed to 0.881 and 0.871, according to the experimental EXAFS fit of Pb foil by fixing CN as the known crystallographic value. * This value was fixed during EXAFS fitting, based on the known structure of Pb. Fitting range: 3.0 ≤ *k* (/Å) ≤ 13.4 and 1.0 ≤ *R* (Å) ≤ 3.0 (Ru foil); 3.0 ≤ *k* (/Å) ≤ 14.0 and 1.0 ≤ *R* (Å) ≤ 4.0 (RuO2); 3.0 ≤ *k* (/Å) ≤ 11.7 and 1.0 ≤ *R* (Å) ≤ 3.5 (211-Ru-3); 3.0 ≤ *k* (/Å) ≤ 14.0 and 1.8 ≤ *R* (Å) ≤ 3.5 (Mo foil); 3.0 ≤ *k* (/Å) ≤ 14.2 and 1.3 ≤ *R* (Å) ≤ 4.0 (MoO3); 3.0 ≤ *k* (/Å) ≤ 13.2 and 1.5 ≤ *R* (Å) ≤ 4.0 (211-Mo-1); 3.0 ≤ *k* (/Å) ≤ 14.0 and 1.5 ≤ *R* (Å) ≤ 4.0 (211-Mo-2); 3.0 ≤ *k* (/Å) ≤ 14.0 and 1.5 ≤ *R* (Å) ≤ 4.0 (211-Mo-3). A reasonable range of EXAFS fitting parameters: 0.700 < *Ѕ*_0_^2^ < 1.000; *CN >* 0; *σ*^2^ > 0 Å^2^; |Δ*E*_0_| < 15 eV; *R* factor < 0.02.

**Table S3.** Comparison of photocatalytic CO_2_ methanation performance with the reported various Mo-containing photocatalysts.

| Photocatalysts | Light source | Catalyst dosage | CO_2_ source | CH_4_ (µmol•g^-1^•h^-1^) | Ref. |
| --- | --- | --- | --- | --- | --- |
| Ru_1_@H-MoO_3-x_ | 300 W Xe (UV−vis−NIR) | 0.02 g | Pure CO_2_ | 27.9 | This work |
| Ru_1_@H-MoO_3-x_ | 300 W Xe (NIR) | 0.02 g | Pure CO_2_ | 9.75 | This work |
| MoO_2_/g-C_3_N_4_ | 300 W Xe (UV–vis–NIR) | 0.05 g | Na_2_HCO_3_ + H_2_SO_4_ | 0.4 | 5 |
| MoS_2_/MoO_3-x_ | 500 W Xe (UV–vis–NIR) | 0.002 g | Pure CO_2_ | 2.03 | 6 |
| MoO_3-x_ | 300 W Xe (UV–vis–NIR) | 0.05 g | Pure CO_2_ | 2.08 | 7 |
| MoO_3-x_/WO_3-x_ | 300 W Xe (UV–vis–NIR) | 0.01 g | Pure CO_2_ | 3.1 | 8 |
| Mo/g-C_3_N_4_ | 300 W Xe (UV−vis−NIR) | 0.1 g | Pure CO_2_ | 6.0 | 9 |
| V_o_-rich MoO_2-x_ | 300 W Xe (UV−vis−NIR) | 0.02 g | Pure CO_2_ | 12.2 | 10 |
| Mo-doped WO_3_·0.33H_2_O | 500 W Xe-lamp (UV–vis–NIR) | 0.025 g | 400 ppm | 5.3 | 11 |
| Bi_2_MoO_6_-OVs | 300 W Xe-lamp (Vis−NIR) | 0.05 g | Na_2_HCO_3_ + H_2_SO_4_ | 2.01 | 12 |
| SnO_2_/Cs_3_Bi_2_Br_9_ | 300 W Xe (UV−vis−NIR) | 0.03 | Pure CO_2_ | 7.13 | 24 |
| Pt/Sr_2_Nb_2_O_7_ | 300 W Xe (UV−vis−NIR) | 0.005 | Pure CO_2_ | 15.65 | 25 |
| NiO/MgAl-LDH | 300 W Xe (UV−vis−NIR) | 0.05 | Pure CO_2_ | 10.50 | 26 |
| Plasmonic Bi_2_WO_6_ | 300 W Xe (UV−vis) | 0.005 g | Pure CO_2_ | 9.95 | 13 |
| Atomically thin CuIn_5_S_8_ layers | 300 W Xe-lamp (Vis−NIR) | 0.005 g | Pure CO_2_ | 8.7 | 14 |
| Co single-atom-site catalysts | 150 W Xe-lamp (UV–vis–NIR) | 0.015 g | Pure CO_2_ | 19.5 | 15 |
| Au/TiO_2_/W_18_O_49_ | 300 W Xe-lamp (Vis−NIR) | 0.001 g | Pure CO_2_ | 0.57 | 16 |
| Ag_2_Cu_2_O_3_ nanowires | 300 W Xe (UV−vis−NIR) | 0.03 g | Pure CO_2_ | 3.55 | 17 |
| Single Pd atoms and Pd nanoparticles on g-C3N4 | 250 W Xe-lamp (UV–vis–NIR) | 0.02 g | Pure CO_2_ | 20.3 | 18 |
| Loading Au@Pd nanoparticles onto V_O_-rich TiO_2_ | 300 W Xe (UV−vis) | 0.002 g | Pure CO_2_ | 26.32 | 19 |
| Mo/g-C_3_N_4_ | 300 W Xe (NIR) | 0.1 g | Pure CO_2_ | 1.9 | 9 |
| V_o_-rich MoO_2-x_ | 300 W Xe (NIR) | 0.02 g | Pure CO_2_ | 5.8 | 10 |
| V_S_-AgInS_2_ nanocrystal | 300 W Xe (NIR) | 0.02 g | Pure CO_2_ | 8.04 | 20 |
| Carbon quantum dots (CQDs)-decorated ultrathin Bi_2_WO_6_ nanosheets | 500 W Xe-lamp (NIR) |  | Pure CO_2_ | 0.4 | 21 |
| CsxWO_3_/g-C_3_N_4_ | 300 W Xe (NIR) | 0.05 g | Pure CO_2_ | 1.72 | 22 |
| CuInSnS_4_ | 300 W Xe (NIR) | 0.05 g | Pure CO_2_ | 5.8 (6.53 μL/h) | 23 |
| MoO_3−x_@ZnIn_2_S_4_ | 300 W Xe (NIR) | 0.02 | Pure CO_2_ | 5.56 | 27 |
| MoO_3−_*_x_* /g-C_3_N_4_ | 300 W Xe (NIR) | 0.05 | Pure CO_2_ | 2.50 | 28 |

**References**

1. G. Kresse, J. Furthmüller, *Comput. Mater. Sci.* **1996,** *6,* 15-50.
2. M. Torrent, N. A. W. Holzwarth, F. Jollet, D. Harris, N. Lepley, X. Xu, *Comput. Phys. Commun.* **2010,** *181,* 1862-1867.
3. J.P. Perdew, K. Burke, M. Ernzerhof, *Phys. Rev. Lett.* **1996,** *77,* 3865-3868.
4. S. Grimme, J. Antony, S. Ehrlich, H. Krieg, *J. Chem. Phys.* **2010,** *132,* 154104.
5. H. J. W. Li, H. Zhou, K. Chen, K. Liu, S. Li, K. Jiang, W. Zhang, Y. Xie, Z. Cao, H. Li, H. Liu, X. Xu, H. Pan, J. Hu, D. Tang, X. Qiu, J. Fu, M. Liu, *Solar. RRL* **2019,** *4,* 1900416.
6. J. Li, X. Xu, B. Huang, Z. Lou, B. Li, *ACS Appl. Mater. Interfaces.* **2021,** *13,* 10047-10053.
7. J. Li, Y. Ye, L. Ye, F. Su, Z. Ma, J. Huang, H. Xie, D. E. Doronkin, A. Zimina, J.D. Grunwaldt, Y. Zhou, *J. Mater. Chem. A* **2019,** *7,* 2821-2830.
8. Y. Liu, X. Dong, Q. Yuan, J. Liang, Y. Zhou, X. Qu, B. Dong, *Colloid. Surface. A* **2021,** *621,* 126582.
9. S. Huang, H. Yi, L. Zhang, Z. Jin, Y. Long, Y. Zhang, Q. Liao, J. Na, H. Cui, S. Ruan, Y. Yamauchi, T. Wakihara, Y. V. Kaneti, Y. J. Zeng, *J. Hazard. Mater.* **2020,** *393,* 122324.
10. X. Wu, W. L. Zhang, J. Li, Q. J. Xiang, Z. Y. Liu, B. Liu, *Angew. Chem. Int. Ed.* **2023,** *62,* e202213124.
11. H. P. Wang, L. Zhang, K. F. Wang, X. Sun, W. Z. Wang, *Appl. Catal. B: Environ.* **2019,** *243,* 771-779.
12. X. L. Yang, S. Y. Wang, N. Yang, W. Zhou, P. Wang, K. Jiang, S. Li, H. Song, X. Ding, H. Chen, J. H. Ye, *Appl. Catal. B: Environ.* **2019,** *259,* 118088.
13. C. H. Lu, X. R. Li, Q. Wu, J. Li, L. Wen, Y. Dai, B. B. Huang, B. J. Li, Z. Z. Lou, *ACS Nano* **2021,** *15,* 3529-3539.
14. X. D. Li, Y. F. Sun, J. Q. Xu, Y. J. Shao, J. Wu, X. L. Xu, Y. Pan, H. X. Ju, J. F. Zhu, Y. Xie, *Nat. Energy* **2019,** *4,* 690-699.
15. J. M. Wang, E. Kim, D. P. Kumar, A. P. Rangappa, Y. J. Kim, Y. X. Zhang, T. K. Kim, *Angew. Chem. Int. Ed.* **2022,** *61,* e202113044.
16. X. Y. Jiang, J. D. Huang, Z. H. Bi, W. J. Ni, G. Gurzadyan, Y. A. Zhu, Z. Y. Zhang, *Adv. Mater.* **2022,** *34,* 2109330.
17. S. M. Deng, R. H. Wang, X. Z. Feng, R. J. Zheng, S. K. Gong, X. H. Chen, Y. Z. Shangguan, L. L. Deng, H. Tang, H. Dai, L. L. Duan, C. Y. Liu, Y. Pan, H. Chen, *Angew. Chem. Int. Ed.* **2023,** *62,* e202309625.
18. P. G. Liu, Z. X. Huang, X. P. Gao, X. Hong, J. F. Zhu, G. M. Wang, Y. E. Wu, J. Zeng, X. S. Zheng, *Adv. Mater.* **2022,** *34,* 2200057.
19. J. X. Fan, L. X. Cheng, Y. N. Liu, Y. F. He, Y. F. Wang, D. Q. Li, J. T. Feng, *J. Catal.* **2019,** *378,* 164-175.
20. K. Wang, H. T. Qin, J. Li, Q. Cheng, Y. F. Zhu, H. Y. Hu, J. Peng, S. Q. Chen, G. H. Wang, S. L. Chou, S. X. Dou, Y. Xiao, *Appl. Catal. B: Environ.* **2023,** *332,* 122763.
21. X. Y. Kong, W. L. Tan, B. J. Ng, S. P. Chai, A. R. Mohamed, *Nano Res.* **2017,** *10,* 1720-1731.
22. J. W. Gu, R. T. Guo, Y. F. Miao, Y. Z. Liu, G. L. Wu, C. P. Duan, W. G. Pan, *Appl. Surf. Sci.* **2021,** *540,* 148316.
23. Y. Chai, Y. H. Kong, M. Lin, W. Lin, J. N. Shen, J. L. Long, R. S. Yuan, W. X. Dai, X. X. Wang, Z.Z. Zhang, *Nat. Commun.* **2023,** *14,* 6168.
24. P. Hu, G. Liang, B. Zhu, W. Macyk, J. Yu, F. Xu, *ACS Catal.* **2023**, *13*, 12623.
25. L. Pan, J. Qi, H. Mei, L. Yao, H. Liu, S. Zhou, G. Zhu, J. Wang, L. Cheng, L. Zhang, *Mater. Today Nano* **2023**, *22*, 100327.
26. H. Yang, H. Hou, M. Yang, Z. Zhu, H. Fu, D. Zhang, Y. Luo, W. Yang, *Chem. Eng.J.* **2023**, *474*, 145813.
27. R. Xiong, X. Ke, W. Jia, Y. Xiao, B. Cheng, S. Lei, *J. Mater. Chem. A* **2023**, *11*, 2178.
28. F. Su, Z. Wang, H. Cao, H. Xie, W. Tu, Y. Xiao, S. Shi, J. Chen, X. Jin, X. Y. Kong, *Catal. Sci. Technol* **2023**, *13*, 1325.
